# Recent Advancements in MXene-Based Biosensors for Health and Environmental Applications—A Review

**DOI:** 10.3390/bios14100497

**Published:** 2024-10-12

**Authors:** Ashraf Ali, Sanjit Manohar Majhi, Lamia A. Siddig, Abdul Hakeem Deshmukh, Hongli Wen, Naser N. Qamhieh, Yaser E. Greish, Saleh T. Mahmoud

**Affiliations:** 1 Department of Physics, United Arab Emirates University, Al–Ain 15551, United Arab Emirates; ashraf.asgerali@ku.ac.ae (A.A.); sanjeetmjh@gmail.com (S.M.M.); lamiaali@uaeu.ac.ae (L.A.S.); abdulhakeem.desh@uaeu.ac.ae (A.H.D.); nqamhieh@uaeu.ac.ae (N.N.Q.); 2 Department of Physics, Khalifa University of Science and Technology, Abu Dhabi 127788, United Arab Emirates; 3 School of Chemical Engineering and Light Industry, Guangdong University of Technology, Guangzhou 510006, China; hongliwen@gdut.edu.cn; 4 Department of Chemistry, United Arab Emirates University, Al–Ain 15551, United Arab Emirates; y.afifi@uaeu.ac.ae

**Keywords:** MXene, MXene-based biosensors, optical biosensors, fluorescence-based biosensors, MXene/MOF composite-based biosensors

## Abstract

Owing to their unique physicochemical properties, MXenes have emerged as promising materials for biosensing applications. This review paper comprehensively explores the recent advancements in MXene-based biosensors for health and environmental applications. This review begins with an introduction to MXenes and biosensors, outlining various types of biosensors including electrochemical, enzymatic, optical, and fluorescent-based systems. The synthesis methods and characteristics of MXenes are thoroughly discussed, highlighting the importance of these processes in tailoring MXenes for specific biosensing applications. Particular attention is given to the development of electrochemical MXene-based biosensors, which have shown remarkable sensitivity and selectivity in detecting various analytes. This review then delves into enzymatic MXene-based biosensors, exploring how the integration of MXenes with enzymes enhances sensor performance and expands the range of detectable biomarkers. Optical biosensors based on MXenes are examined, focusing on their mechanisms and applications in both healthcare and environmental monitoring. The potential of fluorescent-based MXene biosensors is also investigated, showcasing their utility in imaging and sensing applications. In addition, MXene-based potential wearable biosensors have been discussed along with the role of MXenes in volatile organic compound (VOC) detection for environmental applications. Finally, this paper concludes with a critical analysis of the current state of MXene-based biosensors and provides insights into future perspectives and challenges in this rapidly evolving field.

## 1. Introduction

Nanomaterials (NMs), at the forefront of scientific research, have gathered tremendous interest among the scientific community due to their excellent properties and diverse applications. In 1959, Nobel laureate Richard Feynman coined the term nanomaterial for the first time which started from gold sols and developed beyond 2D-nanomaterials in current times [1]. 2D materials are extensively employed in various applications such as biosensors, nanogenerators, resonators, photonics, batteries, supercapacitors, and thermoelectrics. The outstanding properties of nanomaterials such as small size, high surface-to-volume ratio, and optical response are appropriate for their applications in electronics, medicine, energy, environmental remediation, etc. A rapid development of two-dimensional (2D)-based nanomaterials including graphene, dichalcogenides, and MXene has shown an impressive manifold growth owing to their exceptional optical, mechanical, electronic, and physicochemical properties [2]. 2D materials are crystalline solids consisting of multi-element single-atom-thick layers of allotropes or multi-covalent bond complexes [3]. 2D- materials with the invention of graphene, in 2014, have expanded not only to the plumbene family (silicene, phosphorene, antimonene, tellurene, selinene, etc.) but also consist of other materials including WS_2_, MoS_2_, SnSe, black phosphorus, carbon-based NMs, and MXene. To take full advantage of these nanomaterials, it is essential to transform them into devices for identification, characterization, and quantification purposes [4]. This demands the integration of life-science and electronic technology to develop pivotal pathways that understand the bio-molecular interactions with ease since it is crucial in the field of clinical diagnosis and chemical detection. The rapid technological enhancement to satisfy the growing needs of today’s modern society has enabled the development of rapid, viable, and efficient devices for sensing biomolecules called biosensors. Biosensors are effective, lucrative, and onsite detection tools to analyze and detect biomolecules with high sensitivity, repeatability, physicochemical stability, and low detection limit. A biosensor device comprises a bio-recognition component, which refers to the organic counterpart, i.e., enzyme, antibody, and nucleic acid, and a transducer that generates a signal corresponding to the specific interaction. The classification of the biosensor can be made based on either the biorecognition element or the type of transducer. For example, the enzyme-, antibody-, aptamer-, and whole-cell-biosensors are named based on their bio-recognition element. On the other hand, the electrochemical-, optical-, and fluorescence-biosensors are named based on their transducer [5]. Enzymatic biosensors utilize enzymes as biocatalysts, which are efficient at increasing the reaction rate of any biological process. Analyte detection takes place via different mechanisms: (1) The enzyme metabolizes the analyte and thus, the concentration of the enzyme can be determined through the measurement of catalytic transformation of the analyte; (2) the analyte can inhibit or activate enzymes, which provides the opportunity to quantify the analyte via estimating the enzymatic product formation; and (3) by observing the change in enzyme activity.

Electrochemical biosensors rely on the electrochemical properties of both the analyte and transducer. The electrochemical reaction between the bio-recognition element and analyte takes place on the surface of the transducer, which can be measured in terms of voltage, current, impedance, or capacitance. Based on the type of signal from the transducer, they are further classified as potentiometric, amperometric, impedimetric, and conductometric voltammetry [6]. In optical biosensors, the physical or chemical change caused by the bio-recognition element can be detected via a change in the physical properties of the transducer such as polarization, transmission, reflection, refraction, phase, amplitude, or frequency. Both label-based and label-free optical biosensors can be effectively used to quantify analyte by monitoring the optical signal generated through different methods (colorimetric and fluorescence methods) and analyte–transducer interaction, respectively. The fluorescence-based biosensors use fluorescent dyes, quantum dots, and fluorescent proteins as fluorescence probes. The specific fluorescence probe with a high affinity towards the analyte is selected. The interaction between the analyte and probe modulates the fluorescence properties in proportion to the analyte concentration. The interaction with the analytes resulted in the fluctuation of the emission intensity due to different processes, namely, photoinduced electron transfer (PET), Förster resonance energy transfer (FRET), intramolecular charge transfer (ICT), and excited state intramolecular proton transfer (ESIPT) [7].

MXenes, a relatively new class of fastest-growing two-dimensional (2D) transition metal carbides and nitrides, have garnered significant attention in the field of biosensing due to their unique properties and versatile applications. Since their discovery in 2011 by Prof. Yuri Gogotsi [8,9] at Drexel University, USA, MXenes have demonstrated remarkable potential in various fields, including energy storage [10], catalysis [11], electromagnetic shielding [12], and sensing [13]. MXenes are marvelous materials that offer outstanding prospects for transforming into novel products with enhanced technological capabilities [3]. In recent years, the development of MXene-based biosensors has emerged as a promising area of research, particularly for health monitoring and environmental detection applications [14,15]. MXenes possess several advantageous characteristics such as high electrical conductivity, large surface area, excellent hydrophilicity, ease of surface functionalization, and tunable surface chemistry that make them ideal candidates for biosensor development [12]. Moreover, their biocompatibility and stability in aqueous environments further enhance their suitability for biosensing applications [16]. The family of MXenes is generally derived from MAX phases through chemical etching, which leaves behind layered structured MXenes with M_n+1_X_n_T*_x_*. Naguib et al. [17] synthesized Ti_3_C_2_ 2D nanosheets for the first time from the Ti_3_AlC_2_ phase at room temperature using hydrofluoric (HF) acid, which opens the door to synthesize a variety of MXenes via Al etching. This distinctive layered structure offers high surface area, tunable electronic properties, and excellent mechanical strength, satisfying the essential criteria for various applications. A variety of high-quality MAX phases could be created by substituting M, A, and X, resulting in a diverse library of MAX phases available for their conversion into MXenes. Mono- (Ti_3_C_2_T*_x_*) and di-transition metal-substituted MXenes (Mo_2_TiC_2_T*_x_*, Mo_2_Ti_2_C_3_T*_x_*, and Cr_2_TiC_2_T*_x_*) have been created through the etching Al from their corresponding MAX phases. Similarly, two A elements have also been substituted to obtain the Ti_3_(Si,Al)C_2_ MAX phase. Likewise, X can be tuned to obtain MAX phases, e.g., Ti_3_AlCN. To further increase the family of the MXenes, the existing M of any MAX phases has also been substituted with different M partially (Ti_2−y_Nb_y_CT*_x_*, Ti_2−y_V_y_CT*_x_*, and V_2−y_Nb_y_CT*_x_*) to enlarge the family of MXenes [18]. Until now, more than 30 MAX phases have been converted to their respective MXenes among 100 experimentally known MAX phases [9]. MXene materials are also prepared on large scales in laboratory settings resulting in a rapid growth in MXene research [19]. Among the MXenes, Ti_3_C_2_ MXene exhibits a large surface area, high metallic conductivity, high hydrophilicity, and excellent absorption, which makes them favorable for biosensor applications [20]. In addition, the availability of the terminal groups on the MXene surface assists them in interacting with numerous biomolecules through non-covalent interactions, i.e., hydrogen bonds, coordination bonds, van der Waals forces, and electrostatic interactions [21]. Large UV-vis absorption capability along with the possibility of long-range electron and energy transfer makes them ideal candidates for the energy acceptor or quencher species [22].

In the healthcare sector, MXene-based biosensors have shown great promise for the detection of various biomarkers, pathogens, and metabolites [23]. Recent advancements have led to the development of highly sensitive and selective sensors for monitoring glucose levels [24], detecting cancer biomarkers [25], and identifying infectious agents [26]. These sensors offer the potential for rapid, point-of-care diagnostics and continuous health monitoring, which could revolutionize personalized medicine and early disease detection. Environmental applications of MXene-based biosensors have also seen significant progress. Researchers have developed sensors capable of detecting pollutants [15], heavy metals [27], and harmful chemicals in water and air samples with high sensitivity and specificity [28,29]. These advancements contribute to more effective environmental monitoring and pollution control strategies, addressing critical global challenges related to water quality and air pollution. The integration of MXenes with other nanomaterials and advanced sensing techniques has further enhanced their performance and expanded their applications. For instance, combining MXenes with aptamers, enzymes, or antibodies has led to the creation of highly specific and sensitive biosensing platforms [30]. Additionally, the incorporation of MXenes into electrochemical, optical, and field-effect transistor-based sensors has resulted in improved detection limits and response times [31].

In the past, numerous researchers have extensively covered synthesis, characterization, and different application aspects of MXene materials in their review articles. Sinha et al. [32] summed up the electrochemical, gas-absorptive, and piezoresistive sensors’ application. Kalambate et al. [33] briefly described the application of MXene for the detection of non-steroidal anti-inflammatory drugs (NSAIDs). The MXene materials application for the detection of biomarkers and pharmaceutical drugs was presented by Ozcan et al. [34], while the MXene nanocomposite-based electrochemical biosensors by Yoon et al. [26]. Similarly, the review about biomedical (bioimaging, photothermal cancer therapy) [35], photoelectrochemical and electrochemiluminescence sensors [36], physical sensors (pressure, strain, gas, humidity, and temperature sensors) [37], and in-vivo/vitro cancer diagnosis, photodynamic therapy (PDT) [38] could be found in the literature. However, the review specifically focused on the biosensing application of MXene material, especially within the span of the last 5–6 years is scarce. Since MXene materials are seen as promising materials for biosensing applications, their investigation in recent years has also proportionally increased; in contrast, there is a lack of relevant review that summarizes the current advancements. Therefore, in this review article, we have presented recent state-of-the-art and broad prospects of MXene nanomaterials for their application in biosensing. Firstly, the definition and basic properties of MXene along with their preparation methods are discussed with the prime focus on current findings related to the MXene materials for their application in electrochemical, enzymatic, optical, and fluorescence-based biosensors for the detection of key analytes.

## 2. Definition and Characteristics of MXenes

### 2.1. Definition of MXene

MXenes are a class of two-dimensional (2D) inorganic compounds that consist of layers of transition metals, carbides, nitrides, or carbonitrides. They are produced by selectively etching out the ‘A’ element layer from their parent three-dimensional (3D) layered materials called MAX phases [39,40,41,42,43]. MAX phases are represented by the formula $M_{n+1}AX_{n}T_{x}$, where ‘M’ (M = Ti, Mo, Nb, Hf, V, Sc, Cr, Ta, Zr, or W) represents an early transition metal, ‘A’ represents an element mainly from groups 13 and 14, and ‘X’ represents carbon or nitrogen [44]. The resulting MXenes have a general formula of $M_{n+1}X_{n}T_{x}$, where ‘T*_x_*’ denotes surface terminations like –O, –OH, –F, and/or –Cl, which bond to the outer ‘M’ layers after the etching process [45]. The ‘n’ value in the formula can range from 1 to 4, resulting in different MXene structures with varying thicknesses [45]. For instance, M_2_X, M_3_X_2_, and M_4_X_3_ MXenes consist of 3, 5, and 7 atomic layers, respectively [46,47]. These structures are like their MAX phase precursors, exhibiting a hexagonal close-packed (hcp) arrangement with P63/mmc space group symmetry, where ‘M’ sites are closely packed with transition metals, while ‘X’ atoms fill the octahedral sites between the atomic planes [48]. There are a total of 60 experimentally synthesized members of the MAX family formed by using 14 M and 16 A elements; out of them, more than 40 separate thermodynamically stable forms of MXenes have been prepared at lab scale [49]. This diversity arises from the different possible combinations of M, X, n, and T*_x_*, making them a highly versatile class of 2D materials [50].

### 2.2. Synthesis Method and Characteristics

#### 2.2.1. HF Etching Method-Based MXenes and Characteristics

Hydrofluoric acid (HF) was the first etching agent used to synthesize MXenes from their MAX phase precursors. The HF reacts with the Al in the MAX phase, replacing it with surface terminations, such as –F, –OH, and –O, to form the MXene. The synthesis of MXene materials via the HF Etching method is highly efficient in terms of conversion of the MAX phase to MXene; in other words, the HF etches away Al from the MAX phase more efficiently and produces high-quality MXenes. Besides effectiveness, the HF process produces MXene in an easy and cost-efficient manner without the need for bulkier instruments and complicated reaction tools. In addition, the large quantity of MXene preparation is feasible via the HF etching process. However, the HF used in the synthesis of MXene is known for its high toxicity and corrosive properties, posing significant health and safety concerns. This process can be represented by the following chemical reactions: [8,39]

- Ti_3_AlC_2_(*s*) + 3HF(*l*) → AlF_3_(*s*) + 3/2H_2_(*g*) + Ti_3_C_2_(*s*)
- Ti_3_C_2_(*s*) + 2HF(*l*) → Ti_3_C_2_F_2_(*s*) + H_2_(*g*)
- Ti_3_C_2_(*s*) + 2H_2_O(*l*) → Ti_3_C_2_ (OH)_2_(*s*) + H_2_(*g*)
- Ti_3_C_2_(*s*) + 2H_2_O(*l*) → Ti_3_C_2_O(*s*) + 2H_2_(*g*)

This etching process can be used on a variety of MAX phases containing Al, such as Ti_3_AlC_2_, Ti_2_AlC, Ta_4_AlC_3_, TiNbAlC, and Ti_3_AlCN [51]. Additionally, HF can be used to etch non-MAX phases, such as Hf_3_Al_4_C_6_ and Zr_3_Al_3_C_5_. HF etching is influenced by factors such as HF concentration, etching temperature, and etching time. While a 5 wt% HF solution can produce Ti_3_C_2_T*_x_*, higher concentrations of HF (10–30 wt%) can produce Ti_3_C_2_T*_x_* with increased etching efficiency and larger lateral flake size [40]. In contrast, V– and Nb–based MXenes can only be produced using a 50 wt% HF solution. The final MXene product typically has an accordion-like, multilayered structure. To obtain single-layer MXene nanosheets, the synthesized multilayer MXenes need to undergo an intercalation and delamination process, typically using organic chemicals such as dimethyl sulfoxide (DMSO) or tetrabutylammonium hydroxide (TBAOH). This process weakens the van der Waals forces between the MXene layers to separate them. Although effective, HF etching has several drawbacks, including the corrosive, toxic, and hazardous nature of HF, which raises safety and environmental concerns [52].

#### 2.2.2. In Situ HF Etching Method-Based MXenes and Characteristics

In situ HF etching has emerged as a safer alternative to using highly corrosive hydrofluoric acid directly for synthesizing MXenes. This method relies on generating HF in situ through the reaction of an acid with a fluoride salt [53,54]. For instance, a mixture of lithium fluoride (LiF) and hydrochloric acid (HCl) is commonly employed [53]. During the etching process, the acid-fluoride salt mixture selectively removes the A-element layers from MAX phases, leaving behind 2D sheets of transition metal carbides, nitrides, or carbonitrides, known as MXenes [43]. A notable advantage of the in situ HF etching, particularly using the LiF/HCl route, is its ability to directly produce delaminated MXene nanosheets that readily disperse in solutions [40].

One critical factor influencing the characteristics of the synthesized MXenes is the concentration of LiF and HCl in the etching solution. Studies have shown that a 5_M_LiF/6_M_HCl mixture yields Ti_3_C_2_T*_x_* MXene nanosheets with lateral dimensions ranging from 200 to 500 nm. Interestingly, increasing the concentration to 7.5_M_LiF/9_M_HCl leads to significantly larger nanosheets with sizes ranging from 4 to 15 µm. This change in lateral size can be attributed to the higher concentration of LiF, which promotes the intercalation of Li^+^ ions into the interlayer spaces of the MXene structure, facilitating more efficient etching and larger nanosheet formation [54]. Beyond influencing the size, the etching conditions also affect the surface terminations of the resulting MXenes. For instance, using bifluoride salts like NH_4_HF_2_ as the etching agent can lead to MXenes with –OH terminations [55]. However, regardless of the specific in situ HF etching route, the produced MXenes generally possess surface groups such as –F, –OH, and =O [42]. These surface terminations significantly influence the properties of MXenes. For example, a higher proportion of –F groups can reduce the interlayer spacing and decrease water molecule intercalation due to their hydrophobic nature. However, it is important to note that the provided sources primarily focus on the various synthesis methods of MXenes, with less emphasis on the specific characteristics of MXenes produced solely via in situ HF etching.

#### 2.2.3. Synthesis of MQDs and Characteristics

MQDs are typically synthesized using two primary approaches: top–down and bottom–up methods [56,57,58]. Top–down methods involve breaking down larger MXene structures into smaller MQDs using various physical or chemical techniques [56,57]. Common top–down methods include hydrothermal synthesis, solvothermal synthesis, ultrasonication, acid–base reflux, electrochemical methods, and combinations of these techniques. Of these methods, the hydrothermal method is the most widely used. It involves heating an aqueous solution of MXene nanosheets above the boiling point of water, typically between 100 and 200 °C. The size, morphology, and surface terminations of the resulting MQDs can be tuned by adjusting reaction parameters such as temperature, pH, and reaction time. For example, researchers synthesized Ti_3_C_2_T*_x_* quantum dots with average lateral sizes ranging from 2.9 nm to 6.2 nm by varying the hydrothermal reaction temperature between 100 and 150 °C [59].

While top–down methods are prevalent for MQD synthesis, they have drawbacks such as longer reaction times, lower production yields, and the potential use of environmentally harmful chemicals, such as hydrofluoric acid. Bottom-up approaches, which involve assembling MQDs from smaller molecular precursors, offer advantages such as higher atomic utilization, greater control over size and morphology, and the potential for large-scale production. However, research on bottom-up methods for MQD synthesis is still limited. One reported bottom-up method is the pyrolysis method, in which a precursor material containing the desired elements is subjected to high temperatures in an inert atmosphere [56,57].

MQDs possess unique characteristics owing to their small size and quantum confinement effects. They exhibit improved properties compared to their parent MXenes, including better dispersibility, ease of functionalization, and enhanced photoluminescence. MQDs inherit the structural properties of 2D MXenes, typically forming hexagonal lattices [56]. The size of MQDs is a critical factor that influences their properties. Smaller MQDs possess a higher surface area-to-volume ratio, leading to enhanced reactivity and making them suitable for applications such as catalysis and sensing. Additionally, smaller MQDs exhibit a shift in bandgap energy towards the visible region, enhancing their fluorescence properties [56,60].

## 3. MXenes in Wearable Devices

Electronic devices that are both flexible and wearable have tremendously alleviated human life and transformed into a rapidly growing industry in recent times. Wearable electronic devices are robust, lightweight, and durable devices that convert a non-electric physiological activity into electric signals (current/resistance or voltage) [61]. These devices can be used for managing human health and disease diagnosis via detecting important physical and chemical signals of the human body such as blood pressure, heart pulse rate, body temperature, throat moisture, or any other human body information such as joint activity and micro-expressions. Until now, different types of wearable devices have been extensively explored for sensing pressure, strain, and gas molecules in a continuous, onsite, and non-invasive manner [62,63]. For high performance, the electronic device should be able to, on the one hand, capture the body movement of the user and environmental changes including humidity, temperature, etc., and, on the other hand, should be able to cohere to the user skin [64]. The wearable device consists of the circuit board, power supply, electrochemical sensor, analytical transmission system, and user interface. The power supply is an important component of the device, which is a key for the overall stability and longer life of the device [65]. The conventional wearable devices were based on natural conductive cotton fibers and yarns, which are poor electric conductors and photo-insensitive; hence, the cotton fibers and yarns should have to be integrated with numerous other conductive materials [66]. On the other hand, modern wearable sensors are equipped with hydrogels and conductive materials (polymers, carbon-based materials, graphene, MXenes, etc.). MXene materials hold great prospects for their use in wearable devices owing to their biocompatibility, large surface area, high electrical conductivity, and hydrophilic nature and are excellent candidates for fiber-based textiles [67,68].

**Figure 1:**
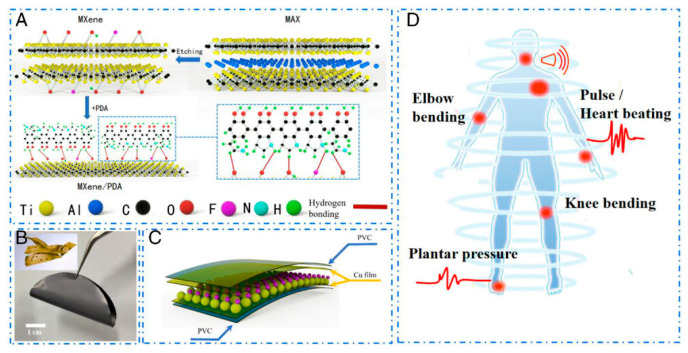
Fabrication schematic diagram of the composite film and flexible pressure sensor. (**A**) The fabrication of MXene/PDA composite film and (**B**) optical image of MXene/PDA composite film. Inset: the microstructure of the MXene/PDA composite film. (**C**) The fabrication of the flexible pressure sensor. (**D**) The application scenarios of the flexible pressure sensor in human health detection. PDA, polydopamine [71].

He et al. [66] used a composite of Ti_3_C_2_T_x_ MXene nanosheets and poly (3,4-ethylenedioxythiophene) polystyrene sulfonate (PEDOT: PSS) as the core. Introduction of PEDOT: PSS with MXene not only enhances the electrical conductivity but also protects MXene from oxidation. The cotton yarn was coated onto the surface of the core to form a core-shell structure that exhibited an electrical conductivity of 21.8 Ω cm^−1^. By using a core-shell structure, a 3D honeycomb-like heater is fabricated. The temperature of the heater reached up to 196 °C at 2.5 V and 64.2 °C at 130 mW cm^−2^ optical density. Chai et al. [68] prepared aerogels using cellulose, MXene nanosheets, and polyurethane composite. MXene nanosheets are evenly distributed in the cellulose matrix to maximize the MXene nanosheets interactions. The aerogel under study showed electromagnetic shielding of 48.59 dB in the X-band with 0.34 S⋅cm^−1^ electrical conductivity. The thermal conductivity of the aerogel reached up to 83.95 mW/(m⋅K) when the aerogels were placed on the surface for 30 min at 120 °C with 60.2 C aerogel surface temperature. The compressive strength and shape recovery of the aerogel were found to be 491.9 kPa and 73%, respectively. The Ti_3_C_2_T_x_ MXene nanosheets have also been used in strain sensor fabrication. Firstly, the self-polymerizable polydopamine (PDA) was coated onto the MXene nanosheets to simultaneously enhance the mechanical properties and protect MXene from oxidation. Secondly, the octadecyl trimethoxy silane (OTMS) was coated onto the PDA@Mxene to make them hydrophobic. Finally, the MXene@PDA@OTMS was immobilized on the cotton to fabricate the strain sensor. The hydrophobic MXene@PDA@OTMS with a contact angle of 156.3 ± 2.4° has achieved a 105 °C temperature at 100 mW/cm^2^ power density [69]. Zhou et al. [70] reported a flexible MXene-Leather composite-based sensor for pressure and joule heating sensing. With a simple vacuum filtration process, the MXene nanosheets were incorporated into the leather via hydrogen bonding. The incorporation of the MXene nanosheets provided a conductive network (24.6 Ω sq^−1^ resistance) and enhanced the mechanical property of the leather, i.e., Young’s modulus up to 9.64 MPa (45.6%). The pressure sensor fabricated using MXene-Leather composite had a sensitivity of 33.58 k/Pa over a wide range of 0.025−12.5 kPa. Han et al. [71] reported the MXene/PDA composite-based wearable pressure sensor, as shown in Figure 1. The addition of the PDA has significantly enhanced the sensitivity of the MXene films from 0.26 k/Pa to 24.7 k/Pa and 138.8 k/Pa for lower- (0.18–2.90 kPa) and higher-pressure (2.90–6.20 kPa) range, respectively. Also, the response and recovery time of the MXene/PDA composite-based sensor were relatively shorter, i.e., <100 ms and <50 ms, respectively.

**Figure 2:**
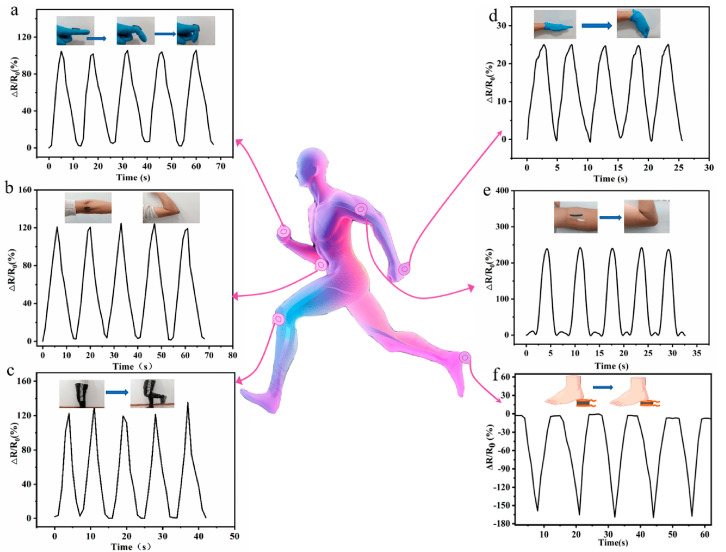
Real-time monitoring of human motions with finger (**a**), elbow (**b**), knee (**c**), wrist (**d**), and arm muscle movements (**e**). (**f**) The real-time monitoring of foot plantar pressure during locomotion [64].

Chen et al. [64] constructed MXene, polyacrylamide, and agar (MXene-PAM/Agar) composite double network structure via a one-pot process for their application in strain sensing. The acrylamide was polymerized in long chains and cross-linked with the MXenes to form a first layered network structure, while another layer was introduced by mixing agar at high temperature to fabricate MXene-PAM/Agar hydrogel. MXene-PAM/Agar hydrogel showed conductivity of 1.02 S/m and 1300% stretchability. The strain sensor could perform well under 80% and 0.35 MPa of compressive strain and stress, respectively. The authors measure the real-time moments of the different body parts, as shown in Figure 2.

Similarly, the sensor could withstand 1000% tensile strain and 0.25 MPa tensile stress. On the other hand, the MXene materials have also been used to fabricate MXene-bacterial cellulose (MXene-BC) supercapacitor [72]. The cross-linking of cellulose bacteria with MXene nanosheets provided a thin film with 88.94 MPa tensile strength, 6.8 GPa Young’s modulus, and 182 S/cm electrical conductivity. The MXene thin films having 10% cellulose bacteria worked well as a supercapacitor electrode and showed an aerial capacitance of 346 mF/cm^2^. MXenes in combination with polyvinyl alcohol (PVA) have also presented better results. By mixing the different concentrations (5–25 wt.%) of the PVA with MXenes, the tensile strength and thermal stability of the MXenes/PVA fibers have been improved [67]. MXenes/PVA fibers-based supercapacitors exhibited the gravimetric and areal capacitance of 119.3 F/g and 130.9 mF/cm^2^, respectively. Fan et al. [73] used the plasmonic silver NPs and polyurethane with MXene nanosheets to build a wearable sensor. The hybridization of the MXene@AgNPs causes enhanced photothermal conversion due to the combined plasmonic effect from AgNPs as well as the photothermal/thermal conductivity effects from MXene nanosheets. The low concentration, about 0.08 wt.%, of plasmonic MXene@AgNPs could produce increased temperature (~111 °C) after applying the 600 mW cm^−2^ light irradiation for 5 min. This increase in the temperature permits the polyurethane thereby healing the damaged coating by 97%. Also, the MXene@AgNPs thin film of ∼60 μm thickness has maintained ∼83% of transmittance. The AgNP@MXene hybrid functions as a highly effective photon captor, energy transformer, and molecular heater due to the amalgamation of (1) ultrahigh photothermal conversion efficiency, high thermal conductivity, and structural properties of MXene; (2) the outstanding plasmonic effect of Ag NPs; and (3) the synergistic effects from their hybrids. The resulting wearable composite coating with ultralow loading of plasmonic AgNP@MXene hybrids (0.08 wt.%wt % or 0.024 vol %) can produce a significant temperature increase of ∼111 ± 2.6 °C after the application of 600 mW cm^−2^ light irradiation for 5 min while maintaining a high optical transmittance of ∼83% at a thickness of ∼60 μm. This local temperature increase can rapidly heal the mechanical damage to the composite coating, with a healing efficiency above 97%.

## 4. MXenes in Volatile Organic Compound (VOC) Detection

In the present modern society, with the growing industrialization, hazardous volatile organic compounds (VOCs) emissions liberated from common pollution sources such as mining, manufacturing, and the automobile industry have become a challenging issue throughout the world. Some of these VOCs (toluene, xylene, phenol, and formaldehyde) at different concentration levels are commonly present in indoor home and office premises, hold neurotoxic and carcinogenic properties, and could yield toxic products via photochemical activation capable of deteriorating human health upon long time exposure [74]. In addition, the majority of the VOCs fall into the category of flammable substances. There is thus a current technological demand for a rapid and reproducible approach to the identification of VOCs [75]. Therefore, advanced sensors are essentially required for fast, sensitive, and accurate detection of hazardous VOC gases for environmental and healthcare applications. Detection of VOCs in parts per million (ppm) level from exhaled breath, which carries ~200 VOCs, is another prominent way to diagnose illness at an early stage [76]. It is known that the ammonia levels in the 50–2000 parts per billion (ppb) levels are critical for the diagnosis of peptic ulcers; similarly, the acetone levels between 300 and 1800 ppb can distinguish healthy and diabetic patients [77]. In literature, numerous oxide-based VOC sensors are reported that work well at high temperatures (300–500 °C), limiting their wide range of applications [74]. Ideally, the sensor for VOCs’ detection should be able to selectively detect the minute concentrations of VOCs, particularly in ppb levels at RT (under ambient conditions) [78]. Also, the VOC detection should be reproducible, recoverable, and cheaper. There are very few VOC sensors working at RT, for example, nickel oxide-based sensors, which can operate at RT but exhibit poor signal-to-noise ratio, sensitivity, and recovery time. The other RT VOC sensors based on graphene and carbon nanotubes need air/oxygen and ammonia supply and current (100 mA) or UV illumination for sensor recovery [74]. Many researchers have investigated the synthesis of 2D MXene materials for their use in VOC sensing.

**Figure 3:**
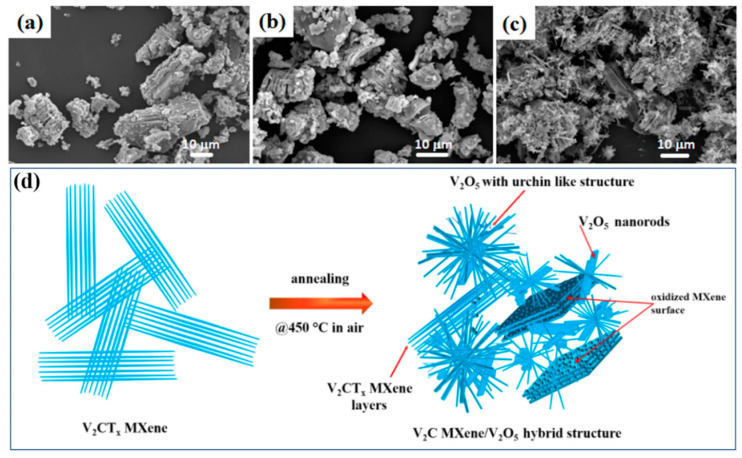
SEM images of V_2_CT*_x_* MXene calcined at different temperatures: 300 °C (**a**), 350 °C (**b**), 450 °C (**c**), and (**d**) schematic diagram of the formation of the V_2_C MXene-derived, urchin-like V_2_O_5_ structure annealed at 450 °C in air [86].

Xu et al. [79] reported Ti_3_C_2_T_x_/SnO_2_ sensors with a large surface area of about 45.186 m^2^/gm for ethanolamine (EA) sensing. The Ti_3_C_2_T_x_/SnO_2_ sensor works well in the 20–600 ppm range, resulting in the sensitive detection of the EA with 76.76 ppb LOD, which is comparably lower than that of the Ti_3_C_2_T_x_ (527.70 ppb), SnO_2_ (187.19 ppb) when used exclusively. The addition of SnO_2_ to the pristine Ti_3_C_2_T_x_ not only enhanced the LOD by 7 times but also showed selectivity for EA among 12 different VOCs. Similarly, to detect the triethylamine (TEA), SnS_2_ nanoflakes were added as a sensitive channel to the Ti_3_C_2_T_x_ MXenes by Han et al. [75]. The SnS_2_/Ti_3_C_2_T_x_ showed 38% response to 50 ppm TEA in 12 s and sensitively detected the TEA in the 2–20 ppm range was 1.2% ppm^−1^. Bhardwaj et al. and Liu et al. [80] investigated the performance of the Ti_3_C_2_T_x_/TiO_2_ for their application in VOC detection. The TiO_2_ was grown in situ because of the self-oxidation of MXene via the hydrothermal method. Obviously, the improved performance of the Ti_3_C_2_T_x_/TiO_2_ as compared to the pristine counterpart was noted. The improved performance of the Ti_3_C_2_T_x_/TiO_2_ is attributed to the interfacial Schottky barrier, which resulted in the improved response for NO_2_ gas up to 1.13 and 2.10–5 ppm (86 times higher than pristine) at RT and 175 °C, respectively. Bhardwaj et al. [81] utilized a SrTiO_3_ with MXenes to fabricate a SrTiO_3_/MXene-based composite sensor for VOC sensing. The authors created a TiO_2_ on MXene by oxidizing the MXene itself at 350 °C for 24 h and then coated it with the SrTiO_3_ moisture-resistant layer. The formation of the TiO_2_ layer has enhanced the response for acetone sensing, for 50 ppm acetone at 150 °C in air, from 175% to 217%. Under 80% relative humid conditions, the SrTiO_3_/MXene showed 68% response for 100 ppb of acetone and LOD of 40 ppb under 150 °C temperature. In another study, single-atom platinum (Pt) was coated onto the Ti_3_C_2_T*_x_* nanosheets (Pt–Ti_3_C_2_T*_x_*) for the detection of TEA. The Pt–Ti_3_C_2_T*_x_* showed better response toward ppb-level at RT. Anchoring of the Pt on MXene not only provided a high specificity but also shortened the short response time in comparison with the pristine Ti_3_C_2_T*_x_*, and thus, an LOD of 14 ppb for detecting TEA was achieved [82]. Doping sulfur (S) atoms into the Ti_3_C_2_T*_x_* MXene has also shown enhanced gas sensing properties as compared to the undoped Ti_3_C_2_T*_x_* MXene [74]. S-doped Ti_3_C_2_T*_x_* MXene selectively detects toluene among other VOCs (namely, ethanol, hexane, toluene, and hexyl-acetate) with higher response for toluene than the undoped Ti_3_C_2_T*_x_* MXene. With S-doped Ti_3_C_2_T*_x_* MXene, the response of toluene has enhanced from ∼214% (1 ppm) to ∼312% (50 ppm) with a notable response even for 500 ppb of toluene. The gold (Au) and Pt atoms are also coated on the Ti_3_C_2_T*_x_* MXene and their ability to sense NH_3_ gas is evaluated by Nam et al. [83]. It was observed that the 1.92 at% Au (under 5 V) and 0.83 at% Pt (under 3 V) showed good selectivity for NH_3_ gas as well as flexibility upon tilting (1000 times) and bending (5000 times). MXene/MoS_2_ nanocomposites prepared via the ultrasonic exfoliation (HIUE) method with 90% yield and significantly shorter etching time of 3 h have also been shown to sense NH_3_ with 21.1% response to the 100 ppm NH_3_ concentration [84]. Our group recently reported a Ti_3_C_2_T*_x_* highly sensitive MXene-based sensor that responds in quick time (53 s) for the detection of acetone as low as 250 ppb at RT, which holds paramount importance in both environmental monitoring and identifying diabetes patients via their breath analysis. The sensor not only showed good selectivity in comparison with the other gases but was also found to be repeatable and highly stable [85]. Besides Ti_3_C_2_T*_x_* MXene, we have also reported a chemiresistive gas sensor using thermally oxidized V_2_CT*_x_* MXene forming urchin-like vanadium oxide (V_2_O*_x_*) hybrid structures, as seen in Figure 3. The V_2_CT*_x_* MXene displayed a 4.6% response toward 15 ppm of acetone, which improved to 11.9% upon the thermal treatment [86].

Rathi et al. [87] added cation surfactant cetyltrimethylammonium bromide (CTAB) during the exfoliation of Nb_2_CT*_x_*. The addition of CTAB has been shown to enhance NO_2_ gas sensing response by 3-fold from 0.543 ppm^−1^ for Nb_2_CT*_x_* to 1.686 ppm^−1^ for Nb_2_CT*_x_*–CTAB. The Nb_2_CT*_x_*–CTAB sensor is capable of detecting trace-level gas concentration even after 30 days. The double transition metal-based Ti_2_VC_2_T*_x_* MXenes are reported to sense ammonia gas molecules with about 25% of sensor response to 100 ppm ammonia at RT within 4s, and the recovery time found to be 16.2 s [88].

## 5. MXenes in Biosensing

MXenes have attracted considerable interest in the fields of analytical nanoscience and biosensing because of their distinct characteristics. The groups on the surface such as OH, O, and F make MXenes water-loving, allowing them to interact with biomolecules through hydrogen bonding, Van der Waals forces, electrostatic interactions, and binding to ligands. This property makes MXenes excellent candidates as carriers in biosensor device applications [32,89,90]. Importantly, various compositions of MXenes have been shown to be biocompatible and non-cytotoxic, further enhancing their suitability for biomedical applications. This review summarizes the composition and analytical performance of MXene-based biosensors across different categories including electrochemical, optical, and other biosensors. Each subsection highlights specific examples showcasing MXenes’ versatility and effectiveness in biosensor fabrication. These case studies underscore MXenes’ broad applicability in sensing technologies, providing valuable insights and data for researchers in the field of biosensing. Readers can readily access detailed research and measurement data from the summarized tables, facilitating further exploration and development of MXene-based biosensors.

### 5.1. MXene-Based Electrochemical Biosensors

A biosensor is a measurement tool that merges a biological sensing component, like a protein or DNA, with a transducer that transforms the biological reaction into a measurable output. The pioneering electrochemical biosensors, introduced in 1967 for glucose monitoring, continue to play an important part in diabetes management. Usually, an electrochemical biosensor consists of three main parts: a working electrode, a reference electrode, and a counter electrode. The working electrode serves as the main detection element and can be made from different types of nanomaterials to identify certain biomolecules or pathogens. Advancements in electrochemical biosensor technology have been driven by the development of biomimetic and bioactive working electrodes. These improvements, coupled with effective signal amplification strategies, enable the conversion of molecular recognition events into measurable electrical signals, such as current, voltage, or impedance. The effectiveness of an electrochemical biosensor is greatly affected by the structure, surface characteristics, and biological operation of the active electrode. Selecting the right materials for the bioreceptor and transducer is crucial for improving the sensor’s ability to distinguish between different substances and its capacity to convert chemical signals into electrical signals. This careful selection of materials is vital for increasing the precision and dependability of detecting specific analytes.

There is an increasing fascination with using MXenes as bioreceptors or transducers in electrochemical biosensors because of their simplicity in making, creating, and modifying them. MXenes, known for their outstanding compatibility with biological materials, provide a flexible base for incorporating fully operational biomolecules like proteins and DNA into electrochemical bio-interfaces. This characteristic makes them perfect for enabling molecular recognition, electrochemical reactions, and signal enhancement—crucial steps for the efficient operation of electrochemical biosensors. MXenes bring notable benefits to biosensor use, such as a large surface area, superior electrical conductivity, and the capability to establish stable and functional connections with biological entities. These features allow MXenes to improve the sensitivity and selectivity of biosensors, enabling the detection of rare biomarkers and pathogens with great accuracy. Additionally, the unique layered structure of MXenes allows for the easy incorporation of various functional groups, further enhancing their versatility in biosensing applications. One of the key strengths of MXenes is their ability to support a wide range of signal amplification strategies. For example, MXenes can be combined with nanomaterials such as gold nanoparticles or quantum dots to enhance electrochemical signals, thereby improving the detection limits of biosensors. Moreover, MXenes’ excellent electrical conductivity ensures efficient electron transfer, which is critical for the rapid and accurate measurement of analyte concentrations.

Even though MXenes were first found more than 10 years ago and have been applied in many well-known areas, there is still a significant lack of thorough studies that cover the growing applications of MXenes in electrochemical biosensing. Current research has only scratched the surface of MXenes’ potential, and there is a need for systematic studies to fully understand and optimize their use in biosensor technologies. Such studies could explore the integration of MXenes with other nanomaterials, the development of new functionalization techniques, and the application of MXenes in real-world biosensing scenarios. As research continues to evolve, a systematic exploration of MXenes’ potential in this domain could pave the way for new innovations and advancements in biosensor technology. By addressing the existing gaps and exploring novel applications, researchers can unlock the full potential of MXenes, leading to the development of next-generation biosensors with unprecedented sensitivity, selectivity, and reliability.

Lately, materials with two dimensions (2D) have been widely used in creating the components that transmit signals in advanced biosensors. This allows for the accurate identification and measurement of DNA and RNA, which are essential for detecting and quantifying nucleic acids. Materials known as MXenes have shown potential as an alternative due to their distinct characteristics. Nonetheless, to create a dependable foundation for electrochemical detection of DNA using MXene 2D materials, it is important to fully comprehend how MXenes interact with DNA, particularly in the spaces between micro-sized MXene flakes where DNA can fit. MXenes have a large surface area, good electrical conductivity, and strong chemical stability, qualities that make them ideal for use in biosensors. Their capacity to establish robust connections with DNA can improve the sensitivity and accuracy of biosensors. However, the interaction between MXenes and DNA needs to be carefully studied to ensure that the structural integrity and biological activity of DNA are maintained during the sensing process. One critical aspect is the intercalation of DNA within the interlayer spaces of MXene flakes. This intercalation can influence the electrochemical properties of the biosensor, potentially enhancing the signal transduction and amplification capabilities. However, it is essential to elucidate how this intercalation affects the stability and functionality of the DNA. Studies should focus on assessing the binding affinity, structural changes, and potential denaturation of DNA when in contact with MXenes. Furthermore, the biocompatibility of MXenes needs to be evaluated to ensure that they do not induce any adverse effects on the DNA or other biological components used in the biosensors. This includes examining potential cytotoxicity, oxidative stress, and any unintended interactions with other biomolecules. Ensuring biocompatibility is paramount for developing reliable and effective MXene-based DNA biosensors for practical applications in medical diagnostics, environmental monitoring, and biotechnology.

**Figure 4:**
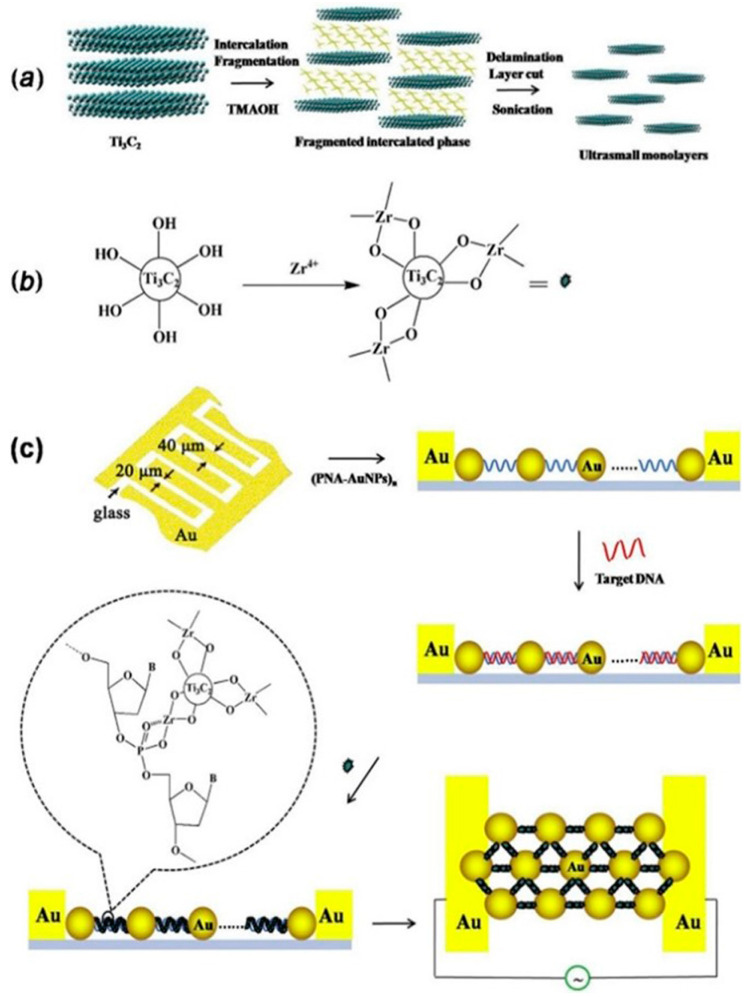
A detailed summary of the process and use of Ti_3_C_2_ MXenes nanosheets in the biosensor for quick identification of *Mycobacterium tuberculosis*: (**a**) the creation of Ti_3_C_2_ MXenes nanosheets, (**b**) the initial treatment of Ti_3_C_2_ MXenes nanosheets with ZrOCl_2_, and (**c**) the design approach of the sensor for swift detection of *M. tuberculosis*. Reproduced from Ref. [89] with permission from Elsevier, Copyright 2020.

Jialin Zhang et al. [89] reported the development of an electrochemical biosensor mediated by conductive Ti_3_C_2_ MXenes for detecting *Mycobacterium tuberculosis*. The biosensor targets the 16S rDNA strands, which contain highly conserved sequences across different species, suitable for general bacterial detection, as well as species-specific variable regions for precise species typing. In this study, a specific fragment of single-stranded DNA (ssDNA) from the species-specific variable region of the 16S rDNA of *M. tuberculosis* was selected as the target biomarker. The biosensor employs peptide nucleic acid (PNA) as the binding agent. PNA is a synthetic version of DNA, made up of a repeating sequence of neutral N–(2–aminoethyl) glycine units linked by peptide bonds, which also contain nucleotide bases. When the target DNA sequence is present, a DNA–PNA complex is formed inside the gold nanoparticle (AuNP) nanogap structure. Then, Ti_3_C_2_ MXenes are bonded to this nanogap structure with a Zr^4+^ cross-linking agent, which can interact with the phosphate groups in the DNA–PNA complex and the hydroxide groups in Ti_3_C_2_ MXenes. This shift from PNA to the conductive Ti_3_C_2_ MXenes within the nanogap structure results in a notable change in electrical conductivity. This shift acts as the basis for the accurate detection of the target DNA sequences, enabling the identification of *M. tuberculosis*. The suggested approach is quick, precise, and capable of detecting as few as 20 colony-forming units per milliliter (CFU/mL) in just 2 h. The biosensor was effectively utilized to identify *M. tuberculosis* in 40 simulated sputum samples, showcasing its real-world utility. Figure 4 provides a comprehensive overview of the preparation and application of Ti_3_C_2_ MXenes nanosheets in the biosensor for rapid detection of *M. tuberculosis*. (a) The Preparation of Ti_3_C_2_ MXenes Nanosheets: The synthesis of Ti_3_C_2_ MXenes nanosheets typically involves etching aluminum layers from the precursor material (Ti_3_AlC_2_) using hydrofluoric acid or a similar etchant.

This process results in the formation of two-dimensional Ti_3_C_2_ MXenes sheets with a high surface area and excellent conductivity. These nanosheets are then exfoliated to obtain a stable colloidal suspension suitable for biosensing applications. (b) The Pretreatment of Ti_3_C_2_ MXenes Nanosheets with ZrOCl_2_: To enhance the biosensor’s functionality, the Ti_3_C_2_ MXenes nanosheets undergo pretreatment with zirconium oxychloride (ZrOCl_2_). This step involves the reaction of Zr^4+^ ions with the hydroxide groups on the surface of the MXenes, facilitating the subsequent attachment of biomolecules. The pretreatment ensures effective cross-linking between the MXenes and the DNA–PNA complex, crucial for the biosensor’s performance. (c) The Constructed Strategy of the Sensor for Rapid Detection of *M. tuberculosis*: The biosensor’s construction integrates the Ti_3_C_2_ MXenes nanosheets into a gold nanoparticle (AuNP) nanogap network electrode.

**Figure 5:**
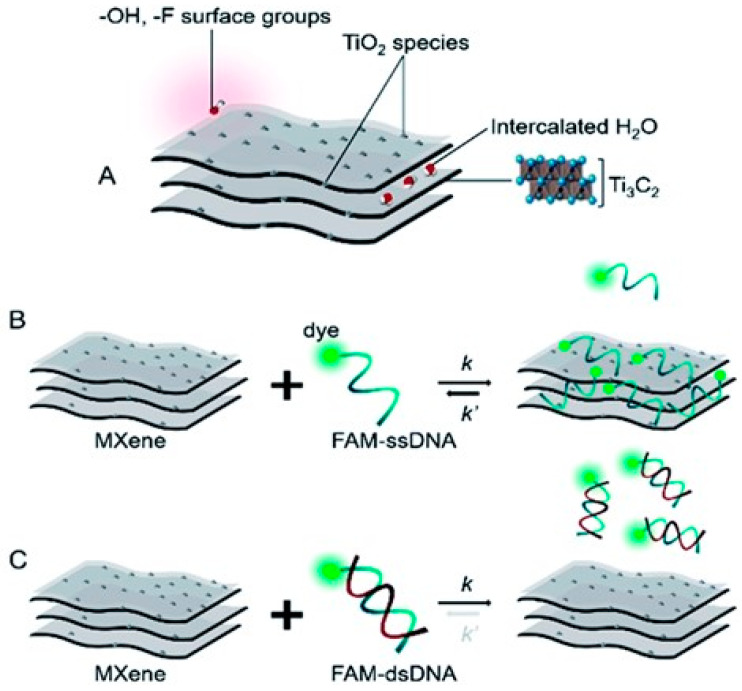
Simplified diagram showing the interaction between MXene (**A**), ssDNA (**B**), and dsDNA (**C**). Reproduced from Ref. [91] under the Creative Commons Attribution-4.0 License (https://creativecommons.org/licenses/by-nc-nd/4.0/), copyright 2019, Royal Society of Chemistry.

C. Lorena Manzanares-Palenzuela et al. [91] conducted a comprehensive study to explore the interaction dynamics between MXene Ti_3_C_2_T*_x_* and fluorophore-tagged DNA using a combination of fluorescence measurements and molecular dynamics simulations. Their research highlighted MXene Ti_3_C_2_T*_x_* as a promising material due to its robust binding affinity toward fluorophore-tagged DNA, enabling highly sensitive biosensing capabilities capable of detecting picomole levels of target molecules with single-base discrimination. The study also uncovered a distinctive kinetic behavior in MXene Ti_3_C_2_T*_x_*, characterized by its ability to effectively trap and release nucleic acids. This behavior suggests potential applications in controlled biomolecule delivery systems, where MXenes could play a crucial role in time-sensitive applications such as drug delivery or molecular sensing with temporal precision. Furthermore, the findings underscore MXenes’ versatility as platforms for nucleic acid interactions, positioning them at the forefront of hybridization–based biosensing technologies. By elucidating the mechanisms behind MXene-DNA interactions at a molecular level, this research contributes to expanding the understanding of MXenes’ role in biomedical applications, including diagnostics, therapeutics, and bioengineering. As shown in Figure 5, the way MXenes and DNA interact is marked by their varying degrees of attraction to single-stranded DNA (ssDNA) versus double-stranded DNA (dsDNA). MXenes usually show a stronger attraction to ssDNA than to dsDNA because they use different, non-covalent ways to bind.

These ways involve van der Waals forces, hydrogen bonds, and π stacking interactions that happen between the phosphate backbone and the nucleobases of DNA. In particular, π–π stacking, which is especially common in MXenes that have sp2 hybridized systems, leads to a stronger bond with ssDNA, making it easier for the unpaired bases to come into contact with the material surface. In contrast, dsDNA, with its rigid double helix structure and bases engaged in intra-strand hydrogen bonding, exhibits weaker adsorption onto MXene surfaces. This differential affinity plays a crucial role in biosensor applications, enabling MXenes to selectively detect specific nucleic acid sequences by leveraging the strong interaction with ssDNA to produce detectable changes in electrical properties, thus enhancing the sensitivity and specificity of MXene-based biosensors.

Jian Yang and his team [92] described a unique 3D-structured setup made of silver (Ag) nanoparticles and Ti_3_C_2_T*_x_*, intended for use in surface-enhanced Raman scattering (SERS) and electrochemical impedance spectroscopy (EIS) biosensors. The combination of Ti_3_C_2_T*_x_* and Ag nanoparticles significantly boosts the Raman signal strength in the Ag/Ti_3_C_2_T*_x_* mixture, rendering it highly effective for SERS purposes. Leveraging the superior SERS capabilities of the Ag/Ti_3_C_2_T*_x_* mixture, along with the magnetic characteristics of Fe_3_O_4_ and the specificity of antigen-antibody interactions, the researchers created a sandwich-like SERS biosensor. This biosensor shows exceptional sensitivity in identifying even small quantities of beta-human chorionic gonadotropin (β-hCG), spanning a wide linear range from 5.0 × 10^−6^ to 1.0 mIU mL^−1^ and a very low detection limit of 9.0 × 10^−7^ mIU mL^−1^. Moreover, Yang and his colleagues developed an EIS biosensor using the Ag/Ti_3_C_2_T*_x_* mixture for the portable detection of β-hCG. This EIS biosensor also displays a wide linear range from 5.0 × 10^−2^ to 1.0 × 10^2^ mIU mL^−1^ and a low detection limit of 9.5 × 10^−3^ mIU mL^−1^. The Ag/Ti_3_C_2_T*_x_* mixture is made by a process called in situ reduction, where silver (Ag) nanoparticles are grown on the Ti_3_C_2_T*_x_* surface through etching Ti_3_AlC_2_ with a solution of HCl and LiF. This synthesis method ensures the integration of Ag nanoparticles onto the Ti_3_C_2_T*_x_* surface, enhancing its electrochemical properties for sensor applications.

For the creation of the electrochemical impedance spectroscopy (EIS) immunosensor designed for portable detection, the setup involves integrating three electrodes within a small electrolytic cell designed by the researchers. The detection of beta-human chorionic gonadotropin (β-hCG) is facilitated using a portable electrochemical workstation (PalmSens4). The immunosensor’s signal is transmitted wirelessly to a mobile phone via Bluetooth for real-time analysis. The foundation of EIS immunosensors is based on the connection of antigens to the Ag/Ti_3_C_2_T*_x_*-based electrodes. This connection raises the resistance of the electrodes in a direct relationship with the amount of the target antigen in the sample. Through monitoring these changes in resistance, the immunosensors can precisely identify and measure β-hCG molecules.

The foundation of EIS immunosensors is based on the connection of antigens to the Ag/Ti_3_C_2_T*_x_*-based electrodes. This connection raises the resistance of the electrodes in a direct relationship with the amount of the target antigen in the sample. Through monitoring these changes in resistance, the immunosensor can precisely identify and measure β-hCG molecules. This method takes advantage of the large surface area and conductivity of the Ag/Ti_3_C_2_T*_x_* mixture, providing sensitive and dependable detection abilities that are ideal for point-of-care diagnostics and medical uses. The practical utility of both immunosensors was validated by successfully detecting β-hCG in actual serum samples, demonstrating satisfactory recovery rates ranging from 98.5% to 102.2%. These results highlight the potential of the Ag/Ti_3_C_2_T*_x_* composite as a versatile platform for developing sensitive and specific immunosensors, promising advancements in clinical diagnostics and biomedical research.

### 5.2. Enzymatic MXene-Based Biosensor

DNAzymes, also referred to as catalytic DNA or deoxyribozymes, are brief DNA sequences that can facilitate different chemical processes. These reactions include DNA phosphorylation, RNA cleavage, and the formation of nucleopeptide linkages, among others [93,94]. The breakthrough in isolating DNAzymes came in 1994 when Breaker and Joyce pioneered their discovery through an innovative in vitro selection method [95]. This pioneering work opened new avenues in nucleic acid research by demonstrating that DNA, traditionally viewed as a carrier of genetic information, can also function as a catalyst akin to enzymes. DNAzymes have since garnered significant attention for their potential applications in biotechnology, medicine, and nanotechnology, owing to their programmable nature and ability to perform precise catalytic functions under diverse conditions.

A wealth of research in the field highlights MXenes and MXene composite materials as capable of maintaining enzyme activity when enzymes are incorporated into their structures. This is attributed to the numerous characteristics and distinctive structural characteristics of MXenes, making them promising candidates for enzyme-based biosensors. Maintaining the function of enzymes is essential for precise and sensitive identification of different substances in medical, environmental, and food safety fields. For instance, Ma and colleagues created an innovative enzyme sensor using Ti_3_C_2_ MXene-coated HRP enzyme complex combined with chitosan. This sensor reached a minimal detection threshold for hydrogen peroxide (H_2_O_2_) and was effectively used to identify small concentrations of H_2_O_2_ in food items [96]. Such applications highlight MXene-based biosensors as promising tools for ensuring food safety and quality control. Similarly, Xu et al. [97] demonstrated the direct integration of Ti_3_C_2_ MXene with HRP enzyme to develop a biosensor specifically designed for detecting H_2_O_2_. The biosensor was utilized to examine blood samples from patients suffering from acute myocardial infarction (AMI) both prior to and following their surgical procedures, demonstrating its capability for use in clinical diagnostics. In summary, the study suggests that MXenes not only maintain the activity of enzymes but also improve the sensitivity and effectiveness of biosensing systems. This quality positions them as useful in various areas, including medical diagnostics, environmental surveillance, and the analysis of food products.

**Figure 6:**
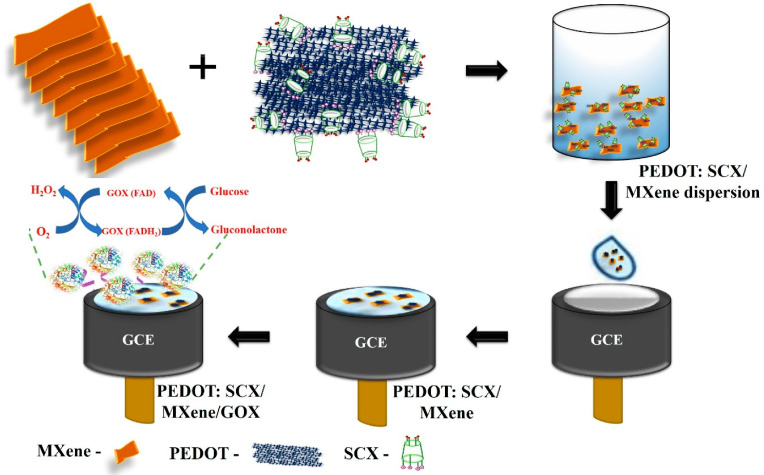
Schematic representation of electrode modification to prepare the glucose biosensor. Reproduced from Ref. [98] under the Creative Commons Attribution-4.0 License (https://creativecommons.org/licenses/by-nc-nd/4.0/), copyright 2022, MDPI.

Besides the applications previously mentioned, MXenes have been thoroughly investigated with various types of enzymes, including glucose oxidase [98], cholesterol oxidase [99], acetylcholinesterase [100], and tyrosinase [101] to detect different molecules. These research efforts highlight the wide-ranging and potent capabilities of MXenes in improving the performance of enzymatic biosensors for a variety of substances. Murugan et al. [100] dispersed MXene in 4-sulfocalix[4]arene (SCX)-doped PEDOT solution to prepare PEDOT: SCX/MXene nanocomposite films, and on top of that the glucose oxidase GOX was immobilized using chitosan (Chit) binder to detect glucose. The nanocomposite PEDOT: SCX/MXene/GOX was then coated on the glassy carbon electrode (GCE), which detects glucose in the 0.5–8 mM range with an LOD of 22.5 µM, as seen in Figure 6. Xia et al. [99] prepared accordion-like Chit/ChOx/Ti_3_C_2_T*_x_* nanocomposite-based enzymatic electrochemical biosensor where the cholesterol oxidase (ChOx) immobilization on MXene. In the process of detection, the cholesterol is oxidized to cholest-4-en-3-one and H_2_O_2_ because of a reaction with ChOx. The levels of generation of cholesterol could be then indirectly estimated by measuring the electro-oxidation generated due to the H_2_O_2_ formation. Under the optimum conditions, the cholesterol can be detected in the linear range of 0.3 to 4.5 nM with a 0.11 nM detection limit and 132.66 μA nM^−1^ cm^−2^ sensitivity.

**Figure 7:**
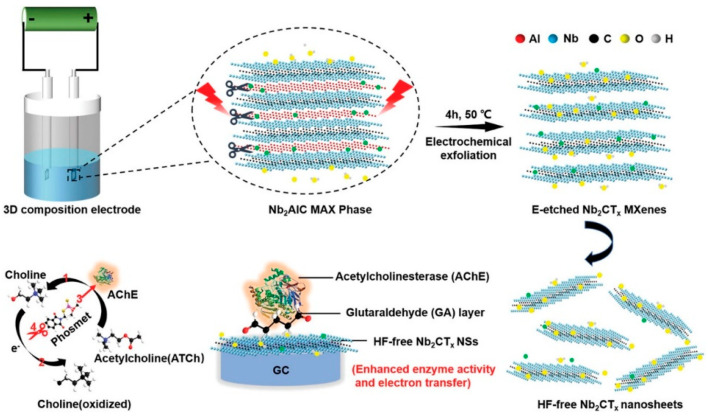
Schematic for exfoliation and delamination process of Nb_2_AlC MAX phase via electrochemical etching and the enzyme inhibition effect for phosmet detection by HF-free Nb_2_CT*_x_*/AChE based biosensor. Reproduced from Ref. [102] under the Creative Commons Attribution-4.0 License (https://creativecommons.org/licenses/by-nc-nd/4.0/), copyright 2020, Wiley-VCH GmbH.

Song et al. [102] investigated electrochemical etching to create a fluorine-free Nb_2_CT*_x_* MXene with minimal toxicity and developed an Nb_2_CT*_x_*/acetylcholinesterase biosensor for the detection of sulfoxide, as shown in Figure 7. This biosensor showed enhanced enzymatic function and electron transfer capabilities compared to those of V_2_C and Ti_3_C_2_ MXene biosensors.

**Figure 8:**
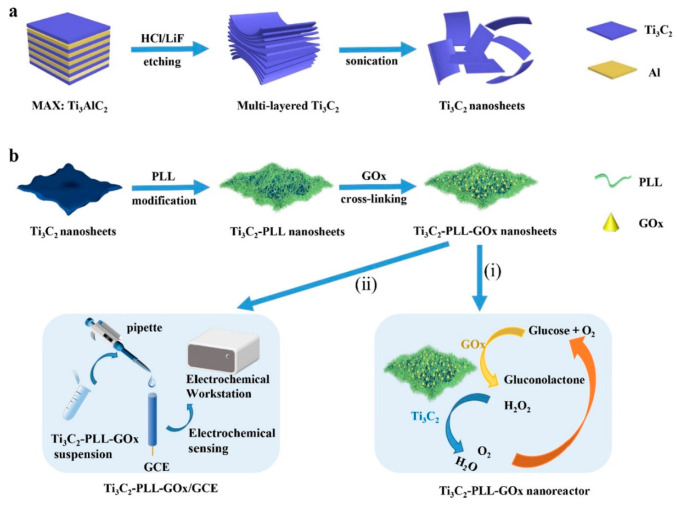
Schematic illustration of the formation of Ti_3_C_2_–PLL–GOx nanoreactor. (**a**) The Ti_3_C_2_ MXene nanosheets were obtained after etching the Al layer from the MAX phase Ti_3_AlC_2_. (**b**) PLL and GOx were sequentially assembled on the Ti_3_C_2_ nanosheets, and the obtained Ti_3_C_2_–PLL–GOx was applied for cascade glucose oxidation (i) and electrochemical glucose sensing (ii). Reproduced from Ref. [103] with permission from Elsevier, Copyright 2021.

Wu et al. [101] also employed Ti_3_C_2_ MXene to attach tyrosinase, enabling direct electron transfer for sensitive and quick detection of phenols. The biosensor they created demonstrated outstanding analytical capabilities over a wide linear range (0.05–15.5 μM) with detection limits as low as 12 nM, proving the potential of Ti_3_C_2_ MXene as a stable and sensitive phenolic biosensor. In recent years, there has been significant exploration into enzyme electrochemical biosensors that offer enhanced efficiency and substrate specificity under mild conditions (refer to Table 1 [103]). Wu et al. [104] created a mixed PLL/Ti_3_C_2_/glucose oxidase biosensor for measuring glucose levels, as presented in Figure 8. This biosensor employs Ti_3_C_2_ MXene to speed up the decomposition of H_2_O_2_ produced during the oxidation of glucose, thus initiating a series of reactions. Furthermore, the Ti_3_C_2_–PLL–GOx nanosheets are deposited on GCE to build a biosensor, which detects the glucose effectively up to 2.6 μM.

These examples highlight MXenes as promising materials for developing advanced enzymatic biosensors with enhanced performance characteristics, including sensitivity, stability, and electron transfer efficiency. Continued research in this area could further expand the scope of MXene-based biosensing device applications in biomedical diagnostics, environmental monitoring, and food safety. The schematic illustration shows the enzymatic inhibition process for detecting sulfoxide using the HF-free Nb_2_CT*_x_*/AChE biosensor, where acetylcholinesterase (AChE) is immobilized on Nb_2_CT*_x_* MXene. This biosensor demonstrates enhanced enzymatic activity and electron transfer compared to other MXene counterparts [102]. These figures highlight the innovative use of MXenes in biosensing applications, showcasing their role in enhancing sensitivity and performance in detecting specific analytes.

### 5.3. Optical Biosensors

Optical biosensors are label-free, tiny, and compact yet powerful portable analytical devices that enable rapid, real-time, and simultaneous detection of multiple analytes (biological and chemical molecules) with high sensitivity, selectivity, and cost-effectiveness. They provide information about a biological interaction via a label-free method via the changes in optical properties of the material near the surface, such as refractive index and polarization, leading to a measurable signal without the need for an external label. Generally, surface-enhanced plasmon resonance (SEPR), interferometry, and waveguide-based biosensors fall into the category of optical biosensors. The research and development of optical biosensors have witnessed exponential growth recently in the fields of healthcare, environment, and biotechnology. The optical biosensors not only provide valuable information on the concentration of the analyte but also shed light on their biochemical interaction at the molecular level with negligible signal-to-noise ratio. Optical biosensors contain a biorecognition element integrated with an optical transducer, which together generates a signal in response to a specific reaction. The biological materials including enzymes, antibodies, antigens, receptors, nucleic acids, cells, and tissues are well-known biorecognition elements that are frequently used in optical biosensors. The analyte is accurately detected in real time with minimal false positive results due to the specific affinity of the biorecognition elements toward the target analyte, facilitating immediate analysis of biological processes for rapid decision-making in medical and research contexts. With their versatility across various biological and chemical sensing tasks, long-term stability, and compatibility with automation, optical biosensors represent a critical technology driving advancements in biomedicine, environmental science, and beyond.

The integration of nanotechnology with advanced materials like MXenes has propelled optical biosensors to new levels of performance and versatility. MXenes, as two-dimensional materials derived from transition metal carbides, nitrides, or carbonitrides, offer several distinct advantages such as high surface area (98 m^2^ g^−1^), superior electrical conductivity (2400 S cm^−1^), biocompatibility, hydrophilicity, and chemical stability in sulfuric acid solution (pH = 1) that enhance the capabilities of optical biosensors. These distinctive properties enable the efficient detection of biomolecules even at extremely low concentrations, which is critical for medical diagnostics and environmental monitoring. The high surface area of MXenes can increase the sensitivity of the biosensor due to the larger number of active sites available for analytes to bind. Moreover, MXenes can be tailored to exhibit tunable optical properties such as absorbance and fluorescence. Owing to their excellent biocompatibility, the MXenes are seamlessly used with biological systems without harming living organisms or clinical samples. Their mechanical robustness and stability under diverse environmental conditions further contribute to the reliability and longevity of biosensing devices.

**Figure 9:**
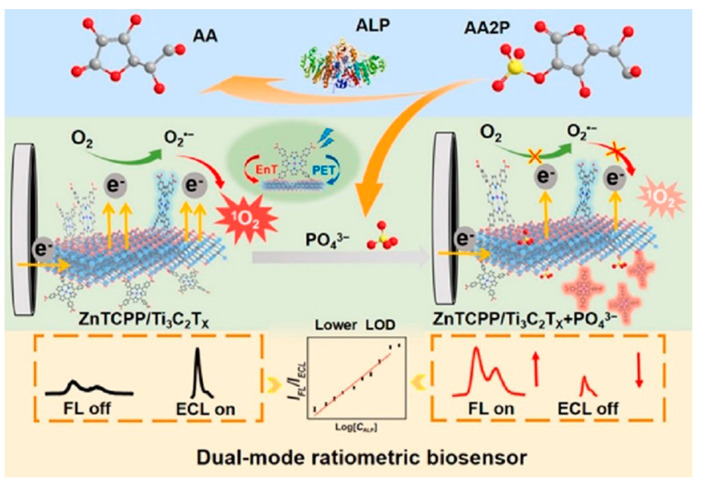
Schematic representation of the dual-mode FL/ECL ratiometric biosensing of ALP. Reproduced from Ref. [108] with permission from Elsevier, Copyright 2024.

Alsaif et al. [106] recently reported a refractive index-based biosensor for the detection of malaria. The sensor comprises a meta-surface with four equal quadrants at the center coated with MXene material and a square-shaped ring resonator coated with black phosphorus which are grown on the surface of the SiO_2_ substrate. At optimum conditions, the sensors exhibit high sensitivity up to 600 GHz/RIU and a low detection limit of 0.109 RIU for malaria. Pisani [4] designed a biosensor consisting of a gold nanoparticle (AuNPs)-functionalized Ti_3_C_2_T*_x_* MXene (AuNPs@Ti_3_C_2_T*_x_*) for detecting human IgG. To detect human IgG, the antihuman IgG was first coated on the AuNPs@Ti_3_C_2_T*_x_*. In the presence of the human IgG, the antibody-antigen interaction takes place which results in the formation of AuNPs clusters. This Au aggregation-induced nanoplatform detects human IgG up to the ≈0.1 ng/mL limit. Singh et al. [107] reported a surface plasmon resonance (SPR)-based biosensor for sensing and differentiating among healthy and tumorous brain tissues based on their unique refractive index (RI). Surface plasmons are generated via Au particles’ coating on the rectangular open channel (ROC) part of the biosensor. Afterward, a thin Ti_3_C_2_T*_x_*–MXene coated over Au particles, and an additional TiO_2_ layer, was placed on the ROC surface to hold the Au particles firmly. The sensitivity values for the gray matter, cerebrospinal fluid, and oligodendroglioma are observed to be 12,352.94, 2030.45, and 672.26 nm/refractive index unit (RIU), respectively. While for the tumorous tissues such as glioblastoma, lymphoma, and metastasis, the sensitivities are 800, 774.9, and 643.26 nm/RIU, respectively. The SPR-based biosensor worked well for the 1.25 × 10^−4^ to 8.09 × 10^−6^ RIU ranges with the maximum figure of merit (FoM) of 126.05 RIU^−1^. Sun et al. [108] investigated the performance of the hybrid dual-mode ratiometric biosensor by combining the fluorescent zinc(II) meso-tetra(4-carboxyphenyl) porphyrin (ZnTCPP) and electro-chemiluminescent Ti_3_C_2_T*_x_* MXene (see Figure 9). The fluorescence of the ZnTCPP is quenched by the Ti_3_C_2_T*_x_* MXene via energy transfer. Through the ZnTCPP/Ti_3_C_2_T*_x_* system, the biosensing of alkaline phosphatase (ALP) is carried out through ALP-catalyzed (PO_4_)^3−^ production in the linear detection range of 0.1–50 mU/mL and detection limit of 0.0083 mU/mL.

**Figure 10:**
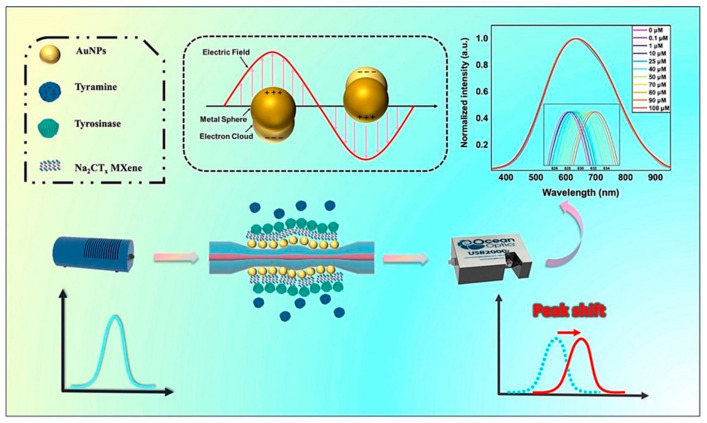
Schematic representation of a WaveFlex biosensor for tyramine detection. Reproduced from [111], with permission from Elsevier, Copyright 2024.

Lang et al. [109] developed a WaveFlex optical fiber biosensor for the detection of Xanthine using a tapered structure consisting of AuNPs and MQDs. The presence of the AuNPs and MQDs generated the SPR to enhance the detection sensitivity. Xanthine was detected via coating xanthine oxidase enzyme on the surface of optical fiber with a 1.93 nm/mM sensitivity, 0–800 mu M detection range, and 146.11 mu M limit of detection. Hasanah et al. [110] proposed a Kretschmann-configured SPR sensor modified by Ti_3_C_2_T_2_ MXene. Both the Ti_3_C_2_T_2_ MXene layers and terminal functional groups are tuned to obtain optimum parameters of MXene. The optimized sensor consisting of six layers of MXene and F_2_ as a terminal group showed the highest sensitivity of 150.131 degrees/RIU with an LOD of 0.01633 RIU. Similarly, as shown in Figure 10, the tyramine is also detected by coating AuNPs and MXene on the fiber surface, which provided the enhanced surface area for tyrosinase enzyme mobilization to sense tyramine with 6.96 mu M detection limit and a sensitivity of 0.0385 nm/mu M [111].

Zhang et al. [112] fabricated flexible W-shaped SPR-based optical fiber biosensor for detecting histamine in food. Besides the AuNPs and Nb_2_CT*_x_* MXene, molybdenum disulfide (MoS_2_) is also coated to improve the surface area and reaction sites. To enhance the specificity, the diamine oxidase (DAO) enzyme is additionally coated on the probe. The proposed biosensor detected histamine in the linear detection range of 0–1000 mu M and achieved 52.5 mu M detection limit along with 4.4 pm/mu M sensitivity [112]. MXene materials have been employed to detect the renal cancer protein biomarkers to diagnose cancer at an early stage. Li et al. [113] designed an optical microfiber coated with Ti_3_C_2_ and AuNPs for the sensing of the carbonic anhydrase IX (CAIX) protein and renal cancer cells with 13.8 zM, 0.19 aM detection limit in pure buffer, and 30% serum solution, respectively. A double S-tapered optical fiber biosensor is reported for tyramine detection. The fiber was coated with AuNPs@GO@tyrosinase and AuNPs@Nb_2_CT*_x_*@tyrosinase to detect tyramine with 17 and 34 pm/mu M in the 0–300 mu M tyramine concentrations range [114].

These studies collectively demonstrate the potential of MXene materials as optical biosensors owing to their versatility, sensitivity, and selectivity for biomedical, environmental, and industrial applications. MXenes materials perform a key role in advancing biosensing technologies through their unique properties and diverse applications, resulting in further enhancements in sensor parameters, along with lower detection limits, enhanced stability, and their ability to integrate into portable devices for on-site and point-of-care detection. MXene materials hold promise to expand the scope and effectiveness of biosensing technologies, addressing diverse analytical challenges, and contributing to advancements in health, environmental sustainability, and food safety.

### 5.4. Fluorescence-Based Biosensors

The fluorescence-based biosensing devices detect the biological interactions via the changes in the fluorescence properties of the fluorophore (fluorescence material) label attached to the analyte or receptor, through well-known methods such as FRET, fluorescent polarization, and dye-based detection. They offer sensitive and selective detection of biological targets by utilizing specific recognition elements such as antibodies or nucleic acids for detecting analytes with minimal interference and high accuracy, beneficial for detecting disease biomarkers, environmental pollutants, and food contamination. The real-time monitoring capabilities of fluorescence biosensors allow rapid analysis. The versatility of fluorescent probes, ranging from organic dyes to advanced nanostructures like quantum dots, further enhances their utility across various applications. These biosensors not only provide quantitative data through measurable fluorescence signals but also support multiplexed detection, allowing simultaneous analysis of multiple targets within a single sample. As technologies advance, integrating fluorescent-based biosensors into portable and miniaturized platforms continues to expand their use in diverse scientific, clinical, and industrial settings, driving innovation in biosensing and analytical chemistry.

MXenes, particularly titanium carbide and titanium nitride derivatives, are promising candidates for fluorescence-based biosensors owing to their exceptional properties such as high surface area, excellent conductivity, and biocompatibility. MXenes can not only serve as a support for immobilizing biomolecules like enzymes or antibodies due to their large surface area and ability to form stable interfaces with biological molecules but also could be integrated with fluorescent probes or quantum dots to enhance signal detection. MXene and fluorophores integration leads to improved sensitivity and signal-to-noise ratios, which is crucial for detecting low analyte concentrations. This technology holds significant potential in medical diagnostics, environmental monitoring, and biotechnology, offering advantages such as high sensitivity, real-time monitoring capabilities, and the potential for multiplexed detection.

#### 5.4.1. Heavy Metal Ion Detection

**Figure 11:**
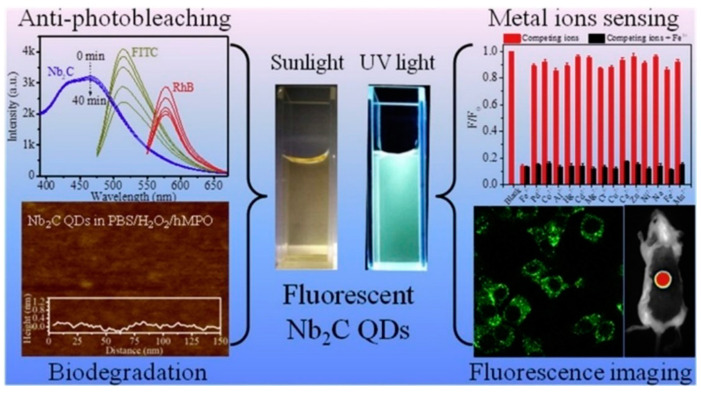
Performance of the Nb_2_C MQDs in the different applications. Reproduced from [109], with the permission from Elsevier, copyright 2020.

Numerous MXene-based optical biosensors dealing with the detection of different types of metal ions via different mechanisms have been reported in the literature. Xue et al. [115] reported photoluminescent Ti_3_C_2_ MXene quantum dot (MQD) with 10% quantum yield (QY) and their potential application in multicolor cellular imaging probes via RAW264.7 cells and Zn^2+^ detection. The comparison of synthesis temperatures and the PL of the MQDs because of the Förster resonance energy transfer (FRET) was declined selectively for Zn^2+^, while the PL was unchanged for other metal ions such as Fe^3+^, Co^2+^, Ni^2+^, Cr^2+^, Pb^2+^, Al^3+^, Cu^2+^, Mn^2+^, and Sn^2+^. Guan et al. [116] prepared N– and P–functionalized Ti_3_C_2_ MQDs with the 2.93 nm size and 20.1% PLQY. N/P@MQDs nanoprobes were employed for the Cu^2+^ ions detection. The PL intensity of the N/P@MQDs was quenched linearly due to FRET upon the addition of Cu^2+^ ions in the 2–100 μM and 250–5000 μM ranges, providing 2 μM of sensitive Cu^2+^ detection. Ti_3_C_2_T*_x_* MQDs are also used to detect Fe^3+^ ions [117]. The white, blue, and blue PL are observed from the Ti_3_C_2_T*_x_* MQDs prepared by the solvothermal method in dimethyl sulfoxide (DMSO), dimethylformamide (DMF), and ethanol solvents, respectively. The solvent has also affected the PL QY (10.7, 6.9, and 4.1%) and average size (3.3, 2.5, and 1.8) of the Ti_3_C_2_T*_x_* MQDs for DMF, ethanol, and DMSO, respectively. Various ions, i.e., Fe^3+^, Fe^2+^, Ni^2+^, Ag^+^, Al^3+^, Co^2+^, Cd^2+^, Mg^2+^, Cu^2+^, Mn^2+^, Hg^2+^, Zn^2+^, and Pb^2+^, are used to check the metal ion sensitivity of Ti_3_C_2_T*_x_* MQDs probe. It was observed that the Ti_3_C_2_T*_x_* MQDs probe selectively detects Fe^3+^ within a good linear relationship range of 5–470 and 510–750 μM and the lowest detection limit of 2 μM. In another study, the Ti_3_C_2_T*_x_* MODs are blended with CsPbBr_3_ halide perovskite to form a nanocomposite-based turn-on sensor for Cd^2+^ detection. The PL intensity of the CsPbBr_3_ has been significantly reduced by Ti_3_C_2_T*_x_* MQDs due to the charge transfer from CsPbBr_3_ to Ti_3_C_2_T*_x_* MQDs. The quenched PL was then linearly recovered with the addition of the Cd^2+^ ions. The detection range for the Cd^2+^ ions was estimated to be from 9.9 × 10^−5^ to 5.9 × 10^−4^ [118]. Yang et al. [119] reported niobium-based Nb_2_C MQDs for the detection of Fe^3+^ ions (Figure 11). The PL intensity of the Nb_2_C MQDs probe quenched linearly (static quenching) with an increase in the Fe^3+^ concentration from 0 to 300 μM, which is attributed to the coordination interaction between the –OH and –COOH surface group present on the Nb_2_C MQDs and Fe^3+^ ions. The Nb_2_C MQDs probes detect the Fe^3+^ ions as low as 0.89 μM.

**Figure 12:**
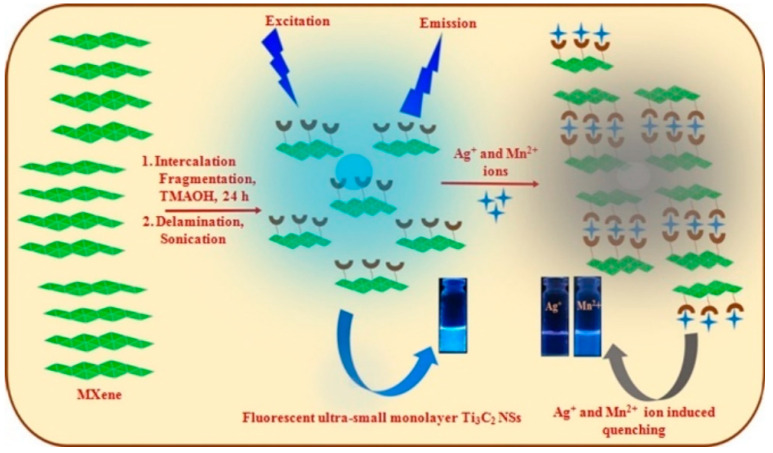
Schematic representation of the Ti_3_C_2_ nanosheets for the detection of Ag^+^ and Mn^2+^ ions. Reproduced from [121], with the permission from Elsevier, copyright 2019.

Desai et al. [120] reported a sensing platform comprising Ti_3_C_2_ nanosheets for the detection of Ag^+^ and Mn^2+^ ions based on PL quenching of Ti_3_C_2_. The hydroxyl– and fluorine–terminated Ti_3_C_2_ nanosheet shows affinity toward Ag^+^ and Mn^2+^ ions, which enables the selective sensing of Ag^+^ and Mn^2+^ ions even from the mixture of other competitive metal ions in environmental and food (tomato, rice, and spinach) samples (Figure 12). The Ti_3_C_2_ nanosheets-based nanoplatform detected the Ag^+^ and Mn^2+^ ions with the detection limit as low as 9.7 and 102 nM within the linear range of 0.1–40 µM and 0.5–60 µM, respectively.

Zhang et al. [121] utilized Ti_3_C_2_ MQDs of 1.75 nm particles with 7.7% quantum yield for the detection of Fe^3+^. The PL quenching in Ti_3_C_2_ MQDs was observed because of the IFE as a result of the redox reaction between Ti_3_C_2_ MQDs and Fe^3+^. Fe^3+^ can be effectively detected up to 310 nM limit in the 5–1000 μM range.

#### 5.4.2. Detection of Biomolecules

MXene nanomaterials are widely used for the detection of biomolecules for clinical diagnostics in the field of biomedical research, where precise detection of biomolecules is essential. Xu et al. [122] prepared Ti_3_C_2_ MQDs using the hydrothermal method for the detection of intracellular glutathione (GSH), an important health and disease biomarker. The Ti_3_C_2_ MQDs produced a blue emission that arises from the surface defects and quantum confinement effects. In the presence of the GSH, the FRET occurs from Ti_3_C_2_ MQDs to GSH for the wide concentration range (1–100 μM), thereby detecting GSH up to 0.02 μM. Shi et al. [123] reported another method to detect GSH using luminescent Cu nanocrystals (NCs) functionalized Ti_3_C_2_T*_x_* MXene flakes. The blue emission of Ti_3_C_2_T*_x_* MXene flakes was quenched by the neighboring Cu NCs through IEF. When the GSH is added to the MXenes–Cu NCs composite, the quenched emission is recovered due to the specific interaction that took place between glutathione and Ti_3_C_2_T*_x_* MXene flakes. The Ti_3_C_2_ MQDs (∼4.2 nm in size) are also used as a probe for detecting alkaline phosphatase (ALP) activity and embryonic stem cell (ESC) recognition. Owing to the overlap between the emission of Ti_3_C_2_ MQDs and the absorption spectrum of p-nitrophenol (p-NP), the energy transfers from Ti_3_C_2_ MQDs to p-NPs via IFE and detect ALP activity with 0.02 U L^−1^ limit of detection [124]. Liu et al. [125] investigated the Ti_3_C_2_ MQDs for cytochrome c detection. The deprotonated Ti_3_C_2_ MQDs exhibit a blue emission around 415 nm with a quantum yield of 22%. In the presence of cytochrome c, the intensity of the Ti_3_C_2_ MQDs has declined linearly in the 0.2–40 μM range with a 20.5 nM detection limit because of the IFE.

**Figure 13:**
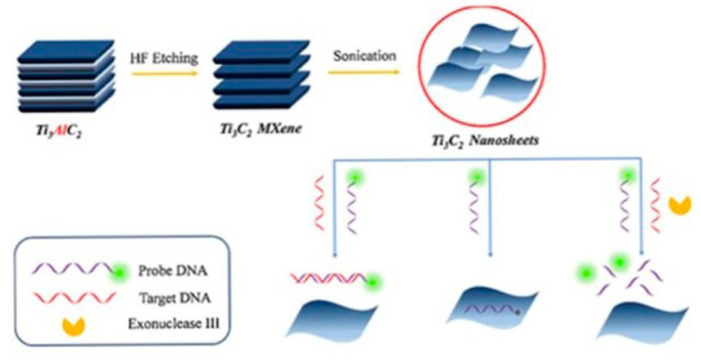
Illustration of the detection of HPV-18 DNA using Ti_3_C_2_ MXene, leveraging the affinity difference between single-stranded and double-stranded DNA on ultra-thin Ti_3_C_2_ MXene for sensitive detection. Reproduced from [129], with the permission from Elsevier, copyright 2019.

To detect an important biomarker, i.e., uric acid, GSH-functionalized Ti_3_C_2_ MQDs are also reported. In a process, the uric acid was first oxidized using uricase enzyme into the allantoin and hydrogen peroxide, whereas o-Phenylenediamine (OPD) was oxidized to the yellow-colored 2,3-diaminophenazine (oxOPD) by using horseradish peroxidase enzyme. The GSH@Ti_3_C_2_ MQDs emit effectively at 425 nm while the oxOPD product produces 568 nm emission. When the uric acid is added to the GSH@Ti_3_C_2_ MQDs and oxOPD composite, FRET takes place between them causing the 425 nm of GSH@Ti_3_C_2_ MQDs to decrease and 568 nm of oxOPD to increase. Using this nanoplatform, the uric acid is detected in the linear range of 1.2–75 μM and a detection limit of 125 nM [126]. In another study by Zhu et al. [127], Ti_3_C_2_ nanosheets were integrated into FRET-based assays with rhodamine B (RhB)-labeled phospholipids for detecting phospholipase D, achieving a sensitivity of 0.10 UL^−1^. The emission of the Ti_3_C_2_ nanosheets is strongly quenched by the neighboring RhB via FRET. Phospholipase D activity cleaves phospholipids, causing the RhB-labeled phospholipids to detach from Ti_3_C_2_ MXenes, which results in fluorescence recovery. Zhu et al. [128] developed a glucose sensor by using Ti_3_C_2_ MXene nanosheets and red-emitting carbon dots (RCDs). Ti_3_C_2_ nanosheets passively quenched the fluorescence intensity of RCDs (>96%) through the IFE, which is recovered upon interaction with glucose. Glucose oxidase catalyzed the oxidation of glucose along with the generation of H_2_O_2_, which further oxidized Ti_3_C_2_ to Ti (OH)_4_, thereby inhibiting IFE from RCDs to Ti_3_C_2_ MXene nanosheets. As presented in Figure 13, the Ti_3_C_2_ MXene-based sensor was also used to detect human papillomavirus HPV-18 DNA [129]. The dye-coated single-stranded (ss) DNA was used as a probe. When the Ti_3_C_2_ nanosheets were coated on the dye@ssDNA probe, the fluorescence of the dye was quenched, whereas the hybridization of the dye-labeled ssDNA with complementary target DNA forms dsDNA, which is responsible for the fluorescence recovery, thereby achieving a low detection limit up to 100 pM. Ti_3_C_2_ MXenes have been explored in complex biological interactions.

**Figure 14:**
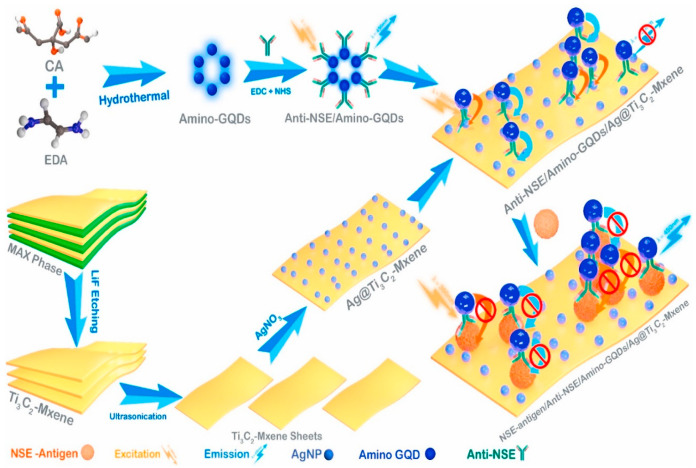
Schematic illustration of anti-NSE/amino-GQDs/Ag@Ti_3_C_2_-MXene-based biosensing platform for fluorometric NSE detection. Reproduced from [21], with the permission from Elsevier, copyright 2022.

Wang et al. [130] investigated the use of chimeric peptide-coated Ti_3_C_2_ MXenes in FRET assays involving histone deacetylase sirtuin-1 and protein phosphatase 2C, showcasing their role in studying molecular interactions critical for understanding cellular mechanisms and disease pathways. Moreover, Ti_3_C_2_ nanosheets have been utilized in pathogen detection, for example, Hong et al. [131] developed a FRET-based sensitive assay for Vibrio parahaemolyticus (VP) detection by using aptamer-modified polyhedral oligomeric silsesquioxane-perovskite quantum dots (POSS-PQDs@Apt) as probe and Ti_3_C_2_ nanosheets as quencher. When the VP is added to the POSS-PQDs@Apt@Ti_3_C_2_ composite, the aptamer selectively binds to the VP leaving behind the Ti_3_C_2_ nanosheets due to the higher binding affinity of the aptamers toward VP than Ti_3_C_2_ nanosheets, which has recovered the quenched POSS-PQDs@Apt emission, thereby achieving a detection limit as low as 30 cfu mL^−1^ in the concentration range of 10^2^–10^6^ cfu/mL. For detecting the endotoxins and *Escherichia coli* pathogens, the Ti_3_C_2_T*_x_* MXenes were integrated with CRISPR–Cas12a by Sheng et al. [132]. The aptamer used in this study initiated the trans-cleavage activity of CRISPR–Cas12a, resulting in the separation of Ti_3_C_2_T*_x_* MXenes from ssDNA to recover the fluorescence, achieving detection limits of 11 pg mL^−1^ for endotoxins and 23 CFU mL^−1^ for *E. coli*. Kalkal et al. [21] reported an Ag/Ti_3_C_2_ composite-based sensor for detecting neuron-specific enolase (Figure 14). In this system, the fluorescence signal from antibody/amino-graphene quantum dots conjugated with Ag/Ti_3_C_2_ is quenched. Upon introduction of the antigen, the fluorescence signal is recovered, demonstrating the capability of the biosensor for sensitive and reproducible detection of neuron-specific enolase (NSE) with 0.05 pg mL^−1^ limit of detection in the 0.0001–1500 ng mL^−1^ detection range. Furthermore, the dual-signal-labeled DNA-functionalized Ti_3_C_2_ MXene nanoprobes were reported for the simultaneous analysis of MUC 1 and miRNA-21 at low concentrations in vitro, alongside in situ imaging of MCF-7 breast cancer cells by Wang et al. [133]. This dual-labeling strategy not only enhances the sensitivity of detection but also enables spatial imaging of biomarkers within cells, providing valuable insights into their expression levels and distribution.

#### 5.4.3. Detection of Miscellaneous Compounds

**Figure 15:**
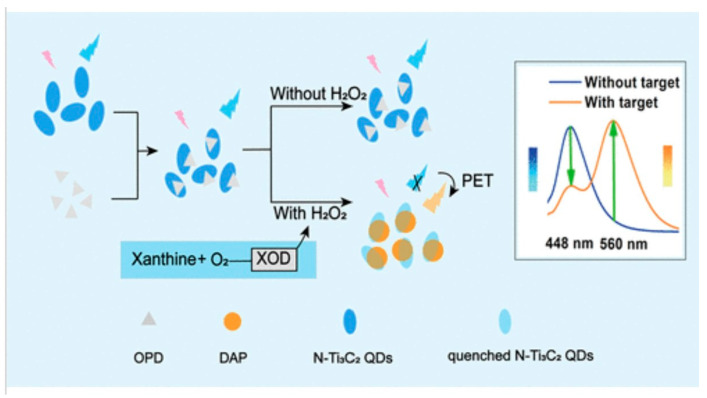
H_2_O_2_ and xanthine detection platform based on N-doped Ti_3_C_2_ MQDs. Reproduced from [136], with the permission from American Chemical Society, copyright 2020.

MXene materials are utilized for the detection of different compounds, for example, Wang et al. [134] prepared uric acid (UA)-functionalized Ti_3_C_2_ MQDs for detecting 2,4,6-trinitrophenol (TNP). The emission of Ti_3_C_2_ MQDs is transferred to the TNP via IFE for a wide linear range of 0.01–40 μM and a detection limit is calculated to be 9.58 nM. The authors employed the UA@Ti_3_C_2_ MQDs for detecting TNP in water as well as on surfaces through a smartphone-based calorimetric method achieving a linear detection range of 10 to 100 ng. MXene materials are also used for detecting an important indicator used for monitoring numerous diseases and cellular health, i.e., intracellular pH. Chen et al. [135] developed a ratiometric fluorescence sensor by combining pH-sensitive polyethyleneimine (PEI)-coated Ti_3_C_2_ MQDs and pH non-sensitive [Ru(dpp)_3_]Cl_2_ for monitoring the intracellular pH changes. Lu et al. [136] used N-doped Ti_3_C_2_ MQDs for the quantitative detection of the H_2_O_2_ and xanthine, see Figure 15. The N-doped Ti_3_C_2_ MQDs were functionalized with 2,3-diaminophenazine (DAP) to build a dual-emissive radiometric sensor for H_2_O_2_ via a photoinduced electron transfer from MQDs to DAP. The composite N-Ti_3_C_2_@DAP nanoprobe could be used to detect H_2_O_2_ and xanthine up to 0.57 μM and 0.34 μM limit, respectively.

**Figure 16:**
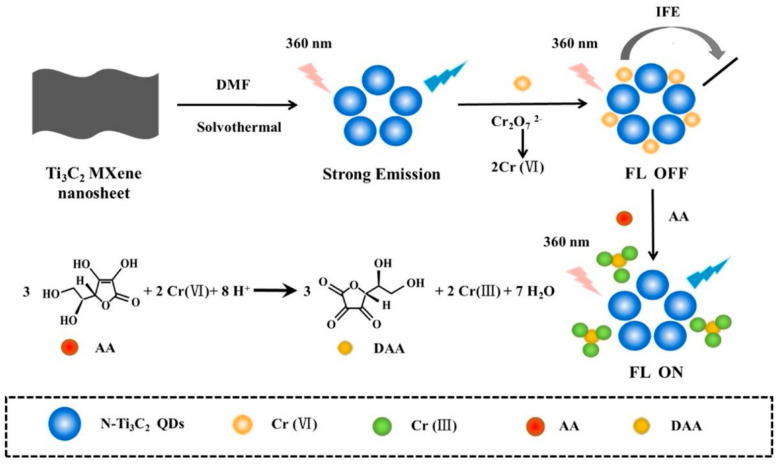
The process of creating intensely luminescent N–Ti_3_C_2_ MQDs and their fluorescence–based approach for detecting Cr(VI) and ascorbic acid. Reproduced with permission from [137], copyright 2024, Elsevier, Copyright 2021.

Similarly, the N-fluorescent Ti_3_C_2_ MQDs have also been employed in the detection of chromium (Cr(VI)) and ascorbic acid (AA) [137]. Figure 16 illustrates the synthesis procedure for the fluorescent N-Ti_3_C_2_ MQDs and the method of detection. The fluorescence of N-Ti_3_C_2_ MQDs is quenched (turn off) by the Cr(VI) via IEF and static quenching, while the addition of AA resulted in the redox reaction between the AA and Cr(VI), which is responsible for the recovery (turn on) of the N-Ti_3_C_2_ MQDs fluorescence. The N-Ti_3_C_2_ MQDs-based nanoplatform performed well in the linear detection range of 0.1–500 μM for both the AA and Cr(VI), with the detection limit of 0.012 (Cr(VI)) and 0.02 μM (AA). Ti_3_C_2_ MQDs have also been used for the detection of curcumin and hypochlorite (ClO^−^). The Ti_3_C_2_ MQDs emission effectively overlaps with the curcumin absorption, causing FRET and thus emission quenching. In the presence of the ClO^−^, curcumin is oxidized to the quinones and recovers the Ti_3_C_2_ MQDs fluorescence. The Ti_3_C_2_ MQDs probe was found to show a linear relationship in the 0.05–10 and 25–275 μM range as well as the detection limit of 20 nM and 5 μM, for curcumin and ClO^−^, respectively [138].

These studies underscore MXenes’ versatility and effectiveness in fluorescence–based biosensing applications, offering robust platforms for detecting enzymes, nucleic acids, and biomolecules with high sensitivity and specificity. The integration of MXenes into optical sensors enhances their capabilities in biomedical research, diagnostics, and cellular imaging, paving the way for advanced analytical techniques in biological and clinical sciences.

## 6. MXene/MOF Composite-Based Biosensors

Metal-organic frameworks (MOFs) are hybrid compounds made from metal ions or clusters (inorganic nodes) and organic linkers. MOFs are crystalline, porous compounds with unique properties, including high surface area, adjustable porosity, and diverse chemical functionalities. These properties open the door for numerous applications, such as chemical detection, energy storage, drug delivery, catalysis, and photocatalysis [139,140,141,142]. Integrating two conductive materials such as MXene and MOFs has enhanced the conductivity and the electrochemical activity due to the swift electron transfer. For instance, anchoring an MOF-based material on MXene layers overcomes the limitations and enhances the poor conductivity and electrochemical surfaces of both materials [143]. Additionally, the incorporation of MXene and MOF provides unique advantages in different applications. These hybrid materials show synergistic effects by combining the unique properties of both materials [143,144,145].

### 6.1. HIV Detection

Human immunodeficiency virus (HIV) is one of the major global health issues that cause acquired immune deficiency syndrome (AIDS) [146]. According to the literature, every year around 1.3 million people die from HIV/AIDS-related diseases. For that, developing reliable and rapid sensors for early detection is critically important [146,147,148].

In 2021, Yunfei Wang and his colleagues successfully prepared an efficient electrochemical luminescence (ECL) biosensor using Ti_3_C_2_T*_x_*/ZIF-8 composite as an ECL emitter to identify HIV-1 protein [149]. By incorporating the imidazole-based-MOF (ZIF-8) into the layered structure of Ti_3_C_2_T*_x_*, the resulting nanocomposite exhibits an enhancement in electrical conductivity and excellent ECL response. The synthesis of Ti_3_C_2_T*_x_*/ZIF-8 composite involves the addition of polyacrylic acid (PPA) to Ti_3_C_2_T*_x_* to avoid MXene sheets agglomeration and to provide a high surface area, enabling ZIF-8 to integrate into an MXene layered structure, leading to rapid electron transfer. The resulting biosensor exhibits high performance, with a low detection limit of 0.3 fM and a linear range between 1 fM and 1 nM, revealing the potential for early and accurate HIV-1 detection [149].

### 6.2. Tyrosine Detection

Tyrosine is one of the important amino acids that helps to maintain nutritional balance in the human body. It plays a role in regulating emotions and stimulating the neurological system because it is found as a precursor to neurotransmitters such as dopamine and thyroxine [150]. Tyrosine is a key component of proteins found in foods like milk, meat, and soybeans [151]. Low levels of tyrosine can lead to conditions such as depression, hypothyroidism, and dementia, while excessively high concentration can contribute to Parkinson’s disease and hyperthyroidism [152,153]. Accurate and fast detection of tyrosine levels can assist in diagnosing these diseases.

**Figure 17:**
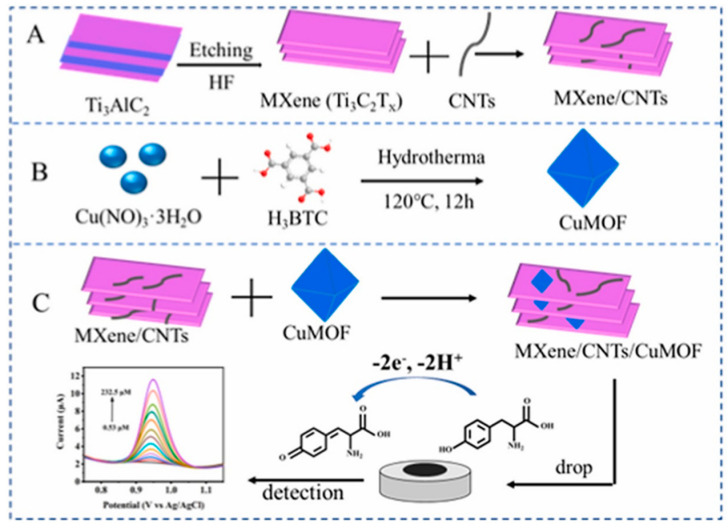
Shows the schematics of MXene/CNT composite (**A**), Cu–MOF (**B**) and MXene/CNT/Cu–MOF (**C**) preparation and tyrosine sensor operation. Reproduced from Ref. [154], with the permission from Elsevier, Copyright 2022.

Lu et al. [154] prepared an electrochemical sensor employing MXene/C nanotubes (CNTs)/Cu–MOF materials for tyrosine detection. The synthesis started by incorporating the CNT into Ti–MXene sheets to expand the space between the layers of the composites and prevent the aggregation of the MXene layers, then octahedral Cu–MOF (Cu–BTC) was added to MXene/CNT composite to enhance the porosity and catalytic performance of prepared material. The obtained SEM images of previous materials (Cu–MOF, MXene, MXene/CNT, and MXene/CNT/Cu–MOF) showed an octahedral structure of Cu–MOF (Cu–BTC), while Ti–MXene has an accordion-like multilayer structure. After the addition of CNTs to MXene, the resulting composite shows a loss of its multilayer structure and the Cu–MOF granules coating the MXene/CNT surface. Prepared MXene/CNT/Cu–MOF composite was layered on GCE to detect tyrosine, as seen in Figure 17. The sensor exhibited a broad linear detection range and low limit of detection (LOD) for tyrosine. It also showed remarkable selectivity, stability, and repeatability with a high rate of recovery and efficient detection of tyrosine, making it suitable for practical use.

### 6.3. Hygromycin B Detection

Aminoglycoside antibiotics (AGs) are antibiotics effective across a broad spectrum of bacterial types, including Gram-positive and Gram-negative bacteria. However, excessive intake of AGs can cause significant harm to the kidneys, brain, and hearing, potentially leading to irreversible organ damage [155,156,157,158]. Although the development of antibiotics over the decades has made AGs less common in modern treatment, they remain a promising area of study due to their potential to address viral and genetic diseases. Detecting aminoglycosides in food quickly and effectively is crucial for ensuring dietary safety and human health. Hygromycin B, an AG with potent antibacterial properties, is commonly used in animal husbandry for the treatment of intestinal nematode infections in pigs and chickens. Like other AGs, Hygromycin B is not metabolized in the body and is excreted in its unchanged molecular form [155,156].

**Figure 18:**
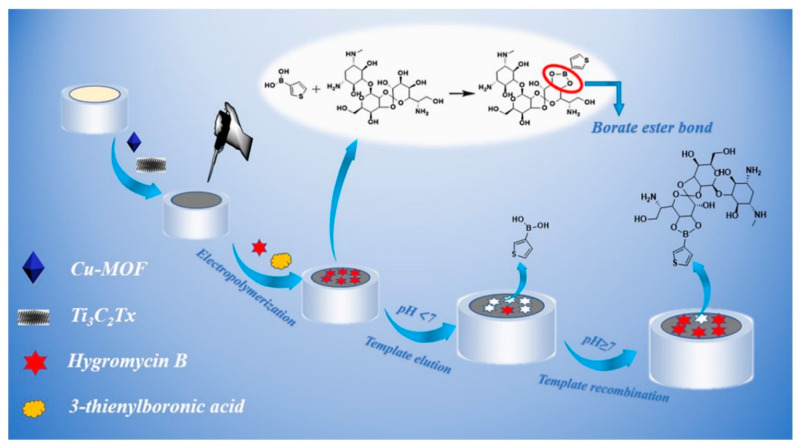
The fabrication process of MIP/Cu–MOF/Ti_3_C_2_T*_x_*/GE sensor for hygromycin B detection in food. Reproduced from Ref. [159], with the permission from Elsevier, Copyright 2022.

Wang et al. [159] successfully fabricated a molecularly imprinted electrochemical sensor (MIES) with Ti_3_C_2_T*_x_* and Cu–MOF (Cu–BTC) to detect hygromycin B in food. The sensor was prepared using the covalently imprinting technology by coating the molecularly imprinted polymers (MIP) film onto the gold electrode surface; during the electropolymerization, the hydroxyl group in the Hygromycin B and the functional monomer (3-theinylboroinc acid) formed the borate ester bond. Cu–MOF was used in the sensor to increase the adhesion surface of the MIP. The electrical conductivity of the fabricated sensor was enhanced by incorporating high-conductivity Ti_3_C_2_T*_x_*, which improved the total sensing efficiency of the MIES. Figure 18 shows the preparation procedure of the MIP/Cu–MOF/Ti_3_C_2_T*_x_*/GE sensor. This prepared sensor exhibits excellent selectivity with a linear range of 5 × 10^9^–5 × 10^6^ M, stability, and reproducibility, which make it suitable for hygromycin B detection in food with a good recovery rate.

### 6.4. CD44 Detection

CD44 is a family of cell adhesion molecules found on different cell surfaces and involved in various biological functions, such as division, cell migration, and cell proliferation [160]. Its expression level is directly connected with the metastasis and progression of cancer, with higher levels in more metastatic tumor cells compared to those with less metastatic potential. Therefore, the CD44 expression level serves as a biomarker for malignant diseases’ detection. For that, reliable and effective CD44 monitoring is a critical focus of cancer research [161,162].

**Figure 19:**
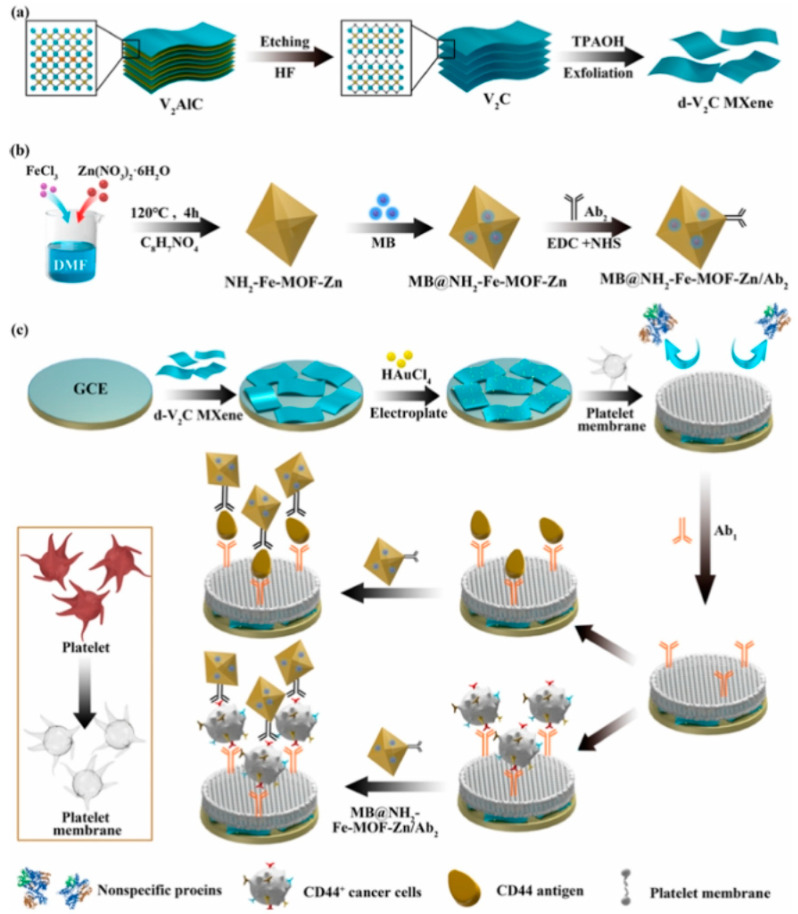
Synthesis and fabrication process of electrochemical immunosensor for CD44 monitoring showing (**a**) d-V_2_C MXene exfoliation, (**b**) MB@NH_2_–Fe–MOF–Zn/Ab_2_, (**c**) assembly of d-V_2_C MXene and MB@NH_2_–Fe–MOF–Zn/Ab_2_ on GCE. Reproduced with permission from Ref. [163], copyright 2022, American Chemical Society.

In 2022, Lian and his colleagues designed a sandwich-type electrochemical sensor for effective CD44 detection [163]. The platform of the sensor was prepared using platelet membrane (PM)/Au NP/V_2_C MXene nanosheet (PM/AuNPs/d-V_2_C)-modified electrode, while methylene blue (MB)/aminated MOF (MB@NH_2_-Fe–MOF–Zn) was used as the signal indicator. V_2_C was used to boost the electrical conductivity of the electrode, and the gold nanoparticles (NPs) were then electrodeposited to enhance the electrochemical properties. The electrode’s surface was then enhanced with the incorporation of anti-CD44 antibodies (Ab1) and poly(melamine) (PM), which prevent nonspecific adsorption and enhance the selective capture of CD44 antigen. Additionally, methylene blue (MB)/aminated MOF (MB@NH_2_–Fe–MOF–Zn) was utilized as a signal indicator to facilitate the covalent binding of anti-CD44 Ab (Ab_2_) through differential pulse voltammetry (DPV). Figure 19 represents the synthesis and fabrication procedure of an electrochemical immunosensor for the detection of CD44. The resulting sensor exhibits an excellent antifouling capability and biocompatibility, making it highly effective to identify the target protein CD44 and detect CD44-positive cancer cells.

### 6.5. Arsenic (III) Detection

The high arsenic (As) level in drinking water leads to significant health risks, like skin diseases, keratosis, and bladder and lung cancer [164]. Arsenic exists in two forms, As (III) and As(V), which are found in drinking water, subsurface water, and surface water. Because of the high toxicity of As (III), the World Health Organization (WHO) proposes that the acceptable concentration of As (III) should be 10 μg·L^−1^ in drinking water and 50 μg·L^−1^ in surface water. Therefore, developing an accurate, sensitive, and rapid method for detecting arsenic in water is crucial [165,166].

Xiao et al. [165] constructed an electrochemical sensor for arsenic monitoring. The sensor was synthesized hydrothermally by depositing a Fe–MOF on the Ti_3_C_2_T*_x_* matrix to prepare the Fe–MOF/MXene electrode, which possesses a large surface area and excellent conductivity. Due to the synergistic interaction between Fe–MOF and MXene, arsenic deposition significantly enhances and boosts the sensing response for As(III) at the Fe–MOF/MXene/GCE interface. This sensing system has achieved high sensitivity and the lowest limit of detection (LOD) at 0.58 ng·L^−1^, compared to previous methods. Moreover, the prepared sensor was successfully utilized to measure As (III) in real water samples with excellent recovery rates and high repeatability, confirming its practical applicability.

### 6.6. Dopamine Detection

Dopamine (DA) is a neurotransmitter that is essential in various biological functions, and imbalances in DA levels can lead to neuroendocrine diseases such as tardive dyskinesia, Parkinson’s, and schizophrenia. Therefore, selective and ultrasensitive DA–detecting methods are essential in diagnostic applications [167].

**Figure 20:**
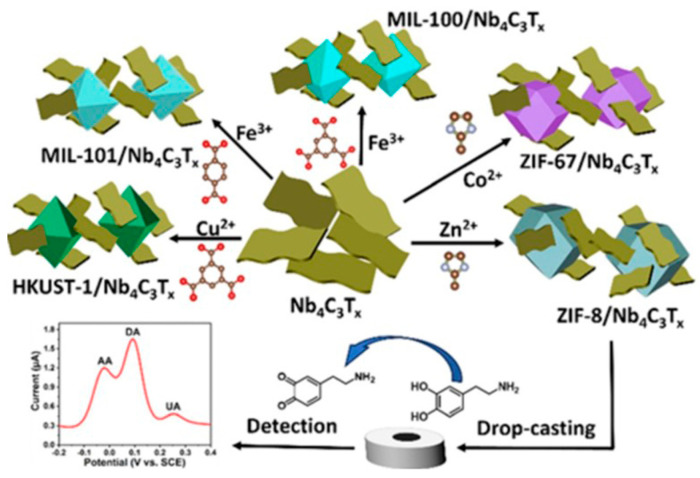
Synthesis and fabrication procedure of electrochemical MOF/Nb_4_C_3_T*_x_* sensor for biomolecules’ detection. Reproduced from Ref. [168] under the Creative Commons Attribution–4.0 License (https://creativecommons.org/licenses/by-nc-nd/4.0/), copyright 2024, Royal Society of Chemistry.

Boruah and his colleague [168] fabricated electrochemical sensor for dopamine detection. They used a simple in situ synthesis method of different MOFs (ZIF–67, MIL–100(Fe), MIL–101(Fe), and HKUST–1) in delaminated Nb_4_C_3_T*_x_* sheets. The high affinity of MXene surface for dopamine, which was further enhanced by the addition of MOFs, leads to superior sensing performance. The MOF/Nb_4_C_3_T*_x_* composites, particularly those with ZIF–8, demonstrated outstanding performance in selectively detecting small biomolecules like dopamine. Figure 20 shows the fabrication of MOF/Nb_4_C_3_T*_x_* composites using different MOFs to detect several biomolecules such as dopamine (DA), ascorbic acid (AA), and uric acid (UA).

## 7. Conclusions and Future Perspectives

MXenes are currently among the most promising materials for a variety of applications including biosensing and health and environmental monitoring. They exhibit unique physicochemical attributes such as high electrical conductivity, biocompatibility, and tunable surface chemistry, which make them highly suitable for biosensing. In this review, we have reviewed thoroughly the recent advancement of MXene-based biosensors encompassing the systematic discussion on the synthesis and characterizations and highlighting their potential in various applications including electrochemical biosensors, enzymatic biosensors, optical-based, and fluorescence-based biosensors. In addition, the role of MXenes as prospective wearable sensors and for VOC detection in environmental applications has been discussed. Their successful integration into these biosensors has demonstrated improved sensitivity, selectivity, and detection limits for a variety of environmental pollutants and biomarkers. Specifically, the ability of MXenes to improve sensor performance by acting as transducers and facilitating electron transfer has opened new avenues for real-time monitoring in medical diagnostics and environmental applications.

Despite these promising developments, challenges that still need attention are improving the large-scale production, reproducibility, and biocompatibility and reducing toxicity and long-term stability of MXene-based biosensors. While many MXene-based biosensors have shown high performance at the lab scale, their scalability for industrial production remains a great challenge. For large-scale production, existing synthesis methods, especially the HF etching procedure, need to be improved. The reproducibility of these sensors in real-world applications also needs more research to ensure consistency across large batches. Researchers must research alternate manufacturing procedures that are scalable and cost-effective, such as green synthesis approaches, without sacrificing MXenes’ inherent properties. Roll-to-roll processing and 3D printing are examples of automated and repeatable manufacturing processes that could provide viable avenues for increasing the supply of sensors. As these challenges are overcome, MXene-based biosensors are poised to play a crucial role in advancing healthcare diagnostics and environmental monitoring technologies.

MXene-based glucose and dopamine biosensors have demonstrated considerable promise, meeting clinical standards for high sensitivity. However, biosensors for environmental monitoring and cancer biomarker detection are still in the development stage and require further improvements in sensitivity and specificity.

In order to improve the performance of biosensors, research should also concentrate on the creation of hybrid MXene composites with MOFs, QDs, or nanoparticles (NPs). These combinations may result in the study of next-generation biosensors with improved sensitivity, the capacity to detect various analytes, and real-time data transfer. The range of detectable analytes for MXene-based biosensors will be expanded by further research into strategic surface functionalization processes, improving their suitability for environmental monitoring and customized treatment. More research should be focused on resolving the scalability of MXene synthesis for their commercialization. Future developments are needed to investigate the employment of MXene-based sensors for point-of-care (PoC) applications for wearable and portable devices. The continuous and non-invasive diagnostics using wearable biosensors, combined with biosensing technologies, artificial intelligence (AI), and the internet of things (IoT) have the potential to further improve personalized health monitoring. This comprehensive review aims to serve as a valuable resource for researchers and practitioners working in the areas of materials science, biosensing, and environmental monitoring.

## Figures and Tables

**Table 1:** Range and LOD values for MXene-based composite materials for different targets.

Composite	Identify Units	Target	LOD	Range	Ref.
Au/Ti_3_C_2_	Glucose oxidase	Glucose	5.9 µM	0.1–18 mM	[105]
PLL/Ti_3_C_2_	Glucose oxidase	Glucose	2.6 µM	4.0–20 µM	[104]
PEDOT: SCX/Ti_3_C_2_T*_x_*	Glucose oxidase	Glucose	22.5 µM	0.5–8 mM	[98]
Ti_3_C_2_/Nafions	Horseradish peroxidase	H_2_O_2_	1 µM	5–8000 µM	[97]
MXene/chitosan	Horseradish peroxidase	H_2_O_2_	0.74 µM	5–1650 µM	[96]
Chit/ChO_x_/Ti_3_C_2_T*_x_*	Cholesterol oxidase	Cholesterol	0.11 nM	0.3–4.5 nM	[99]
Ti_3_C_2_	Tyrosinase	Phenol	12 nM	50 nM–15.5 µM	[101]
CS–Ti_3_C_2_T*_x_*	Acetylcholinesterase	Acetylthiocholine chloride	3 fM	10 nM–10 fM	[100]
GA/Nb_2_CT*_x_*	Acetylcholinesterase	Phosmet	144 pM	200 pM–1 µM	[102]

LOD: limit of detection; µM: micromolar; nM: nanomolar; fM: femtomolar; pM: picomolar.

## Data Availability

Not Applicable.
